# *Streptomyces avermitilis* MICNEMA2022: a new biorational strain for producing abamectin as an integrated nematode management agent

**DOI:** 10.1186/s12866-024-03466-3

**Published:** 2024-09-07

**Authors:** Wafaa H. Radwan, Ahmed A. M. Abdelhafez, Ahmed E. Mahgoub, Mona S. Zayed

**Affiliations:** 1 Department of Microbiology, Faculty of Agriculture, Ain Shams University, Hadayek Shoubra, P.O. Box 68, Shoubra El-Kheima, Cairo, 11566 Egypt; 2 Department of Plant Protection, Faculty of Agriculture, Ain Shams University, Hadayek Shoubra, P.O. Box 68, Shoubra El- Kheima, Cairo, 11566 Egypt; 3 Faculty of Organic Agriculture, Heliopolis University for Sustainable Development, Cairo, Egypt

**Keywords:** ABA, *S. avermitilis*, Spectral analysis, HPLC, LC-MS, Root-knot nematode, Biocontrol

## Abstract

**Background:**

Abamectin (ABA) is considered a powerful insecticidal and anthelmintic agent. It is an intracellular product of *Streptomyces avermitilis*; is synthesized through complicated pathways and can then be extracted from mycelial by methanol extraction. ABA serves as a biological control substance against the root-knot nematode *Meloidogyne incognita*. This investigation is intended to reach a new strain of *S. avermitilis* capable of producing ABA effectively.

**Results:**

Among the sixty actinobacterial isolates, *Streptomyces* St.53 isolate was chosen for its superior nematicidal effectiveness. The mycelial-methanol extract of isolate St.53 exhibited a maximum in vitro mortality of 100% in one day. In the greenhouse experiment, the mycelial-methanol extract demonstrated, for the second-stage juveniles (J_2_s), 75.69% nematode reduction and 0.84 reproduction rate (Rr) while for the second-stage juveniles (J_2_s), the culture suspension demonstrated 75.38% nematode reduction and 0.80 reproduction rate (Rr). Molecular identification for St.53 was performed using 16 S rRNA gene analysis and recorded in NCBI Genbank as *S. avermitilis* MICNEMA2022 with accession number (*OP108264.1*). LC-MS was utilized to detect and identify abamectin in extracts while HPLC analysis was carried out for quantitative determination. Both abamectin B1a and abamectin B1b were produced and detected at retention times of 4.572 and 3.890 min respectively.

**Conclusion:**

*Streptomyces avermitilis* MICNEMA2022 proved to be an effective source for producing abamectin as a biorational agent for integrated nematode management.

**Supplementary Information:**

The online version contains supplementary material available at 10.1186/s12866-024-03466-3.

## Introduction

Nematodes are non-segmented invertebrates that are thought to be the most prevalent animals on earth [1]. Among the nematodes that live in the soil, some play crucial ecological roles in the soil food web by regulating carbon and other nutrient recycling (which increases nutrient availability to plants) [2], while others are deemed phytosanitary risks. Plant-parasitic nematodes (PPN) are among the most harmful plant pathogens worldwide [3], with approximately $173 billion in annual economic losses globally [4].

*Meloidogyne* spp. are the most commercially and scientifically important PPNs because they cause significant economic harm to many host plant species in a variety of conditions [5]. *Meloidogyne incognita*, *M. javanica*,* M. arenaria*, and *M. hapla* represent 95% of all Root-knot nematodes (RKNs) populations [6, 7]. The most commercially significant crops, such as banana, tomato, cowpea, sweet potato, and maize, are being attacked by these species [8]. Plant growth is stunted, and eventually, plant vegetation is damaged due to yellowing, wilting, and other effects [9]. Consequently, plants became susceptible to other pathogens and abiotic stresses [10]. Although chemical control of RKNs by different synthetic nematicides is among the most popular management practices, especially those infest tomato roots, to control the nematodes and enhance plant yields in both open-field and protected agriculture, biological control techniques should be developed as integrated management programs for plant nematodes due to the dangers of potential health hazards and environmental contamination by chemical nematicides [11].

*Streptomyces avermitilis* belongs to actinobacteria, which are prokaryotic microorganisms that are well-known as the producers of a substantial number of primary and secondary metabolites possessing activities against diverse pathogens [12, 13]. *Streptomyces avermitilis* can produce avermectins as antiparasitic compounds which are among the most efficient nematicides [14].

Avermectin is a macrocyclic lactone that possesses the capability to kill infective juveniles and reduce egg hatching [15]. Abamectin is amongst the most recommended bio-rational tools that belong to the avermectin group, which is referred to as avermectin B1. Abamectin is usually a blend of avermectin B1a (≥ 80%) and B1b (≤ 20%) [16]. Abamectin’s constituents B1a and Bl b are nearly identical in their biological and toxicological properties [16].

Although abamectin is considered the most applicable insecticide, nematicide, and acaricide, on vegetables, fruits, and crops, it has revealed low toxicity to beneficial arthropods, which enables its use in Integrated Pest Management (IPM) programs [14, 17].

Currently, the most common commercial forms of avermectin consist of wettable powders, emulsifiable concentrations, aqueous capsule solutions, and water-dispersible granules [18, 19]. However, these formulations have some drawbacks, including excessive use of organic surfactants and solvents, dust dispersion, low efficacy, complex production processes, incomplete release of active ingredients, and high formulation costs. Therefore, the main objective of this research is to produce abamectin from a newly isolated *S. avermitilis* strain, to develop a new crude extract product that is cost-effective for deployment as a new biocontrol agent against *M. incognita.*

## Materials and methods

### Isolation of actinobacterial isolates from soil

Forty soil samples were gathered from nine different Egyptian governorates (Monofiya, Sohag, Giza, Beni Suef, Beheira, Qalioubiya, Ismailia, Fayoum, Gharbiya) during the Fall season of 2020. Soil samples were collected from vegetated soils at a depth of ten centimeters and then packed in sterilized plastic bags, transported to the lab, and stored at 4^o^C for further studies. Isolation was carried out using starch nitrate agar medium and the conventional dilution plate procedure [20]. The Petri dishes were inoculated using the diluted samples, and incubated for fourteen days at 28 °C. Rough colonies that seemed to be actinobacteria were picked up and streaked on starch-nitrate agar [21]. The pure colonies were maintained on starch-nitrate agar medium and stored at refrigeration temperature (4^o^C), in 20% glycerol stocks at -20^o^C, and sub-cultured at monthly intervals. These experiments were conducted at the Microbial Inoculant Center (MIC), Faculty of Agriculture, Ain Shams University, Cairo, Egypt.

### Standard inoculum preparation

Spore suspensions were prepared from the selected actinobacterial isolates by inoculating each isolate on starch nitrate agar medium plates (9 cm in diameter). After fourteen days of incubation at 28ºC, surface growth (sporulation) on the agar plates was scratched in 50 mL of sterile saline water (0.9% NaCl solution) and the final spore concentration was adjusted to 16 × 10^9^ spores mL [22]. To prepare vegetative mycelial inoculum, 5 mL of each selected isolate’s spore suspension was transferred to an Erlenmeyer flask containing 50 mL of seed medium (Yeast malt glucose medium (YMG)) [22]. The flasks were then incubated for 24 h on an orbital shaker at 150 rpm at 28^o^C. After incubation, 5 mL of the vegetative mycelium was utilized to inoculate 50 mL of the production medium and incubated at 150 rpm for ten days at 28 °C [22]. As previously mentioned, this method was used for the cultivation of actinobacterial isolates and the synthesis of secondary metabolites [23].

### In vitro nematicidal potentiality of the selected isolates

The chosen isolates were assessed for their nematicidal activity using cell-free supernatant or methanol-extracted mycelium as follows: isolates were cultivated in the production medium broth for ten days at 28^o^C and then centrifuged at 8000 rpm for 30 min. at 4^o^C.

For mycelial extraction, the mycelial growth of each isolate was gathered and mixed with methanol (99%) at a ratio of 1:9. The mycelia methanol mixtures were then homogenized by ultrasonication at 40 kHz for 30 min., after which the mixtures were centrifuged at 8000 rpm for 30 min. at 4^o^C. Afterward, methanol was removed by rotary evaporator at 45ºC, and the dried extract was re-dissolved in 15 mL (the original volume) of distilled water.

The nematicidal potential of the extracts derived from actinobacterial isolates was examined on *M. incognita* second-stage juveniles (J_2_s) [24] as follows: 1 mL of distilled water containing 200 J_2_s/mL was added to 1 mL of mycelial methanol extract or supernatant extract of each actinobacterial isolate, individually. The control treatment was prepared by adding 1 mL of the medium solution without bacterial isolate to 1 m/L of each nematode suspension containing the same number of nematodes. The number of juveniles, both alive and dead, was counted for each treatment utilizing a light microscope after 24, 48, and 72 h. of the incubation period. Each treatment was prepared with three replicates. The nematodes’ mortality was assessed by observing their straight form and lack of movement after post-stimulation using a fine needle. The mortality of nematodes was computed as follows:

$${\rm{Mortality }}\% {\rm{ }} = {\rm{ }}\left[ {{\rm{ C}}1{\rm{ }}-{\rm{ C}}2{\rm{ }}/{\rm{ C}}1} \right]{\rm{ }} \times {\rm{ }}100$$

Where: C1 is the number of alive nematode larvae in the control.

 C2 is the number of alive nematode larvae in the treatments.

### Efficiency of the selected isolates to control *M. incognita* on tomato plants

The pot experiment was carried out to examine the antinematodal activity of the culture suspension, culture supernatant, and methanol-extracted cells of the most efficient isolates (St.25, St.44, St.48, and St.53). These isolates were selected from the previous experiment for their antagonistic activity towards *M. incognita* under greenhouse conditions. Tomato seeds (*Lycopersicon esculentum* Mill., variety 023, susceptible to *M. incognita*), obtained from SAKATA company, Thailand were sown in sterilized soil at 28 ± 2^o^C. Tomato seedlings with true-stage leaves were transplanted individually into pots of 20 cm diameter filled with 1500 g of sterilized soil. After seven days of transplantation, plants were divided into 5 main groups. The groups were treated according to the following: Control plants without nematode: each plant was treated with 10 mL of water only; control plans with nematode only: ten-milliliter suspension of *M. incognita* containing 100 vigorous J_2_s/mL was used to inoculate each plant; plants treated with culture suspension: ten mL of *M. incognita* suspension and fifteen milliliters of culture suspension obtained from an isolate fermentation broth that was aged for ten days were used to inoculate each plant; Plants treated with culture supernatant: 10 mL of *M. incognita* suspension and fifteen milliliters of culture supernatant obtained from an isolate fermentation broth that was aged for ten days were used to inoculate each plant; and plants treated with mycelial methanol extract: ten milliliters of *M. incognita* suspension and 15 mL of mycelial methanol extract obtained from an isolate fermentation broth that was aged for ten days were used to inoculate each plant. Each treatment was prepared with three replicates, and the pots were kept in the greenhouse for one month (April 2023) and watered daily at an average temperature of 28 ± 2^o^C. Plants were cautiously uprooted after 45 days of treatments.

The roots were divided into two groups. The first group of roots was stained and stored using acid fuchsin in cold lactophenol. The stained roots were immersed in water and sliced into 1 cm sections to count the galls, females, and egg masses. The second group of roots was incubated in tap water to generate J_2_s from egg masses using the reported procedure [25]. The number of nematodes (J_2_s) in the soil was extracted using a sieving and decanting technique [26] and then counted under a light microscope.

The percentages of reduction in nematode parameters were calculated for comparison. The reproduction rate of nematode (Rr) was estimated by dividing the final population of nematode (Pf) by the population of initial nematode (Pi). Plant growth criteria including shoot length (cm), fresh and dry shoot weights (g), and root fresh weight (g) of tomato were measured. Numbers of leaves and flowers were measured. The most efficient isolate, causing the highest mortality or inhibition, was selected for identification by phenotypic and genotypic characteristics.

### Phenotypic features of the selected actinobacterial isolate

Morphological and cultural characteristics as well as the reaction to Gram staining of the selected isolate were examined using light and scanning electron microscopy [27].

### Physiological and biochemical characteristics of the selected actinobacterial isolate

The physiological and biochemical features of the selected isolate were determined as follows:

The capability to produce melanoid pigment was assessed [28] by inoculating the selected isolate on tyrosine agar and peptone-yeast extract iron agar media. Evaluations were conducted to determine the occurrence of the pigment after 4 days of incubation at 28 °C. The ability to grow on Czapekʾs agar medium [29] was tested by inoculating the selected isolate on the agar plate at 28 °C, for 14 days [29]. Tolerance to sodium chloride was tested by inoculating the isolate on an inorganic salt starch agar medium supplemented with (4, 7, 10, and 13%) NaCl and incubated at 28 °C, for 14 days. The antibiotic sensitivity test of the actinobacteria isolates against streptomycin sulfate (50 µg/mL) was tested in Bennet’s agar medium [30] using a disc diffusion technique.

Production of amylase was tested by inoculating the selected isolate on starch agar medium and exposing it to 3 mL of 1% iodine after 14 days of incubation at 28 °C. A clear zone encircling the colonies indicated the capability of the isolate to produce amylase [31]. Lipase production was examined by inoculating the isolate on a solid tributyrin agar medium [32]; after ending the incubation period, plates were flooded with 3 mL of 1% CuSO_4_. Lipase synthesis was noticed *via* the appearance of a greenish-blue color along the colonies’ borders [33]. The production of gelatinase was tested by inoculating the selected isolate on a nutrient gelatin medium [34] and then incubating it at 28 °C for 7 days. After the incubation period had ended, all the tubes were refrigerated for 15 to 30 min. at 4 °C. Gelatinase activity was confirmed due to liquefaction [35]. Cellulase production was assessed by inoculating the isolate on a carboxy methyl cellulose medium [36]. Plates were flooded with 0.25% Congo red for 15–20 min. once the incubation phase had ended, then rinsed with 1 M NaCl for 15 min. The development of a clear zone revealed the production of cellulases [37].

The capability of the selected actinobacterial isolate to utilize 10 different carbon compounds as a sole carbon source, (D-glucose, D-xylose, L-arabinose, L-rhamnose, D-fructose, D-galactose, raffinose, D-mannitol, L- inositol, maltose, and sucrose) was examined by the reported method [38]. Each carbon source was added to the medium at a proportion of 1% w/v. The selected isolate was inoculated on the salt starch agar medium by streaking across the plate medium’s surface, which included one of the selected carbon sources, and then the growth of the isolate was determined after an incubation period of 14 days at 28 °C.

### Identification of abamectin B1b and B1a by LC-MS

LC-MS analysis was conducted on LTQ-Orbitrap (Thermo Scientific, model: LC 2040 Controller). Chromatography was carried out on a Dikma C18 column (5 μm, 250 × 4.6 mm) with a mobile phase of solvent A: B (solvent A: water; solvent B: acetonitrile), flow rate of 0.2 mL/min. The wavelength scan range was from 200 to 800 nm. The mass charge scan range was from 200 to 2000 m/z. The data-dependent mass spectrometry method was employed and all the top five ions of each peak were fragmented by collision-induced dissociation at 35 eV [39].

### Quantification of abamectin’s B1a and B1b by HPLC

Abamectin’s B1a and B1b standard solutions with a final concentration of 2 mg/mL were prepared as stock solutions and diluted to 0.052, 0.104, and 0.208 mg/mL respectively. The absorption area of the standard solutions was measured at 245 nm and a standard curve was constructed using the equation: Y = 772155x – 246,785; R² = 0.9994 for abamectin B1a and Y = 17793x – 6437; R² = 0.9981 for abamectin B1b. All trials were carried out in triplicates. The standard curve and the absorption area of each sample were used to calculate the yields of abamectin, B1a, and B1b [39].

The culture suspensions of the selected isolate (mycelium and supernatant**)** were diluted ten times (v/v) in methanol and then homogenized by ultrasonication at 40 kHz for 30 min. The precipitates were discarded by centrifugation at 12,000 rpm for 30 min and the supernatant was further filtered by a 0.22 μm filter further filtered the supernatant. Samples were injected into a Shimadzu HPLC instrument equipped with a PDA detector (SPD-M20A). Chromatography was carried out on a Dikma C18 column (5 μm, 250 × 4.6 mm) with a mobile phase of solvent A: B = 88: 12 (solvent A, 0.1% formic acid in acetonitrile; solvent B, 0.1% formic acid), with a flow rate 1.0 mL/min. HPLC traces were recorded by monitoring the absorption at 245 nm.

### Molecular Identification of the selected actinobacterial isolate

The selected isolate was identified by the 16 S rRNA gene sequence analysis method as follows: the isolate’s genomic DNA was isolated and the polymerase chain reaction (PCR) was performed to amplify the 16 S rRNA gene sequence using the two universal primers (F1: 5, AGAGTTT (G/C) ATCCTGGCTCAG 3, and R1 5, ACGG (A/C) TACCTTGTTACGACTT 3). Purification of the PCR product was completed using a QIA quick gel extraction kit (Qiagen, Germany). The genomic 16 S rRNA gene sequencing of the purified PCR product was conducted by Macrogen, Inc., South Korea. BLAST searches were completed using the NCBI server [40]. The phylogenetic tree was generated using the neighbor-joining cladogram and maximum-parsimony algorithms using the MEGA 11 program [41].

### Molecular docking

Molecular docking was performed using CDOCKER protocol in Accelrys Discovery Studio^®^ 2.5. The docking study was conducted to investigate the interactions between abamectin (B1a and B1b), isolated from the *S. avermitilis* MICNEMA2022, and the crystal structure of C. elegans GluCL (glutamate-gated chloride channel) receptor [42]. The receptor protein file was retrieved from the RCSB Protein Data Bank (PDB). Ligands (either standard inhibitors (ivermectin or test compounds) were docked into the active sites of GluCL using Schrodinger software 9.3 (Schrodinger Sofware Solutions, USA). The procedure involved the following critical steps, (i) ligprep, (ii) protein preparation wizard, (iii) glide grid generation, and (iv) docking. The grid was generated in proximity to the active sites and docking was performed using the Glide (grid-based ligand docking energetic) module of Schrodinger 9.3. Interactions were visualized using Incentive PyMOL viewer (v1.8.2.3). Abamectin was docked into the active sites of GluCL using Schrodinger software 9.3 (Schrodinger Sofware Solutions, USA). The process included the following steps: (i) ligprep, (ii) protein preparation wizard, (iii) glide grid generation, and (iv) docking. The grid was generated near the active sites, and docking was performed using the Glide (grid-based ligand docking energy) module of Schrodinger 9.3. The interactions were visualized using Incentive PyMOL viewer (v1.8.2.3).

### Statistical analysis

The obtained data were statistically analyzed using one-way ANOVA and LSD 5% tests at a level of significance of *P* < 0.05 using the Costas program (Version 6.400) [43].

## Results

### Isolation and morphological characterization of actinobacterial isolates

Forty soil samples were gathered from nine governments, resulting in a total of sixty actinobacterial isolates at different geographical locations in Egypt, during the fall season of 2020. All isolates were purified and screened for their Gram stain to determine the morphology of the mycelium. The results showed that all isolates were Gram-positive with developed branching, non-fragmented aerial mycelium bearing long non-motile spore chains that were not carried in verticillate sporophores. In a color series, the percentage of color produced by these isolates as soluble pigments varied as follows: red 4%, brown 8%, green 18%, white 20%, and grey 50%. Based on the color of their aerial mycelium, the actinobacterial isolates were divided into five groups (Grey, white, green, brown, and red). Based on the previous results, these isolates were classified as actinobacteria.

### The nematicidal potentiality of the obtained actinobacterial isolates

Aside from the confirmation measurements, the effectiveness of the selected isolates as microbe-derived nematicidal agents against plant-parasitic nematodes *M. incognita* second-stage juveniles (J_2_s) was evaluated.

**Fig. 1:**
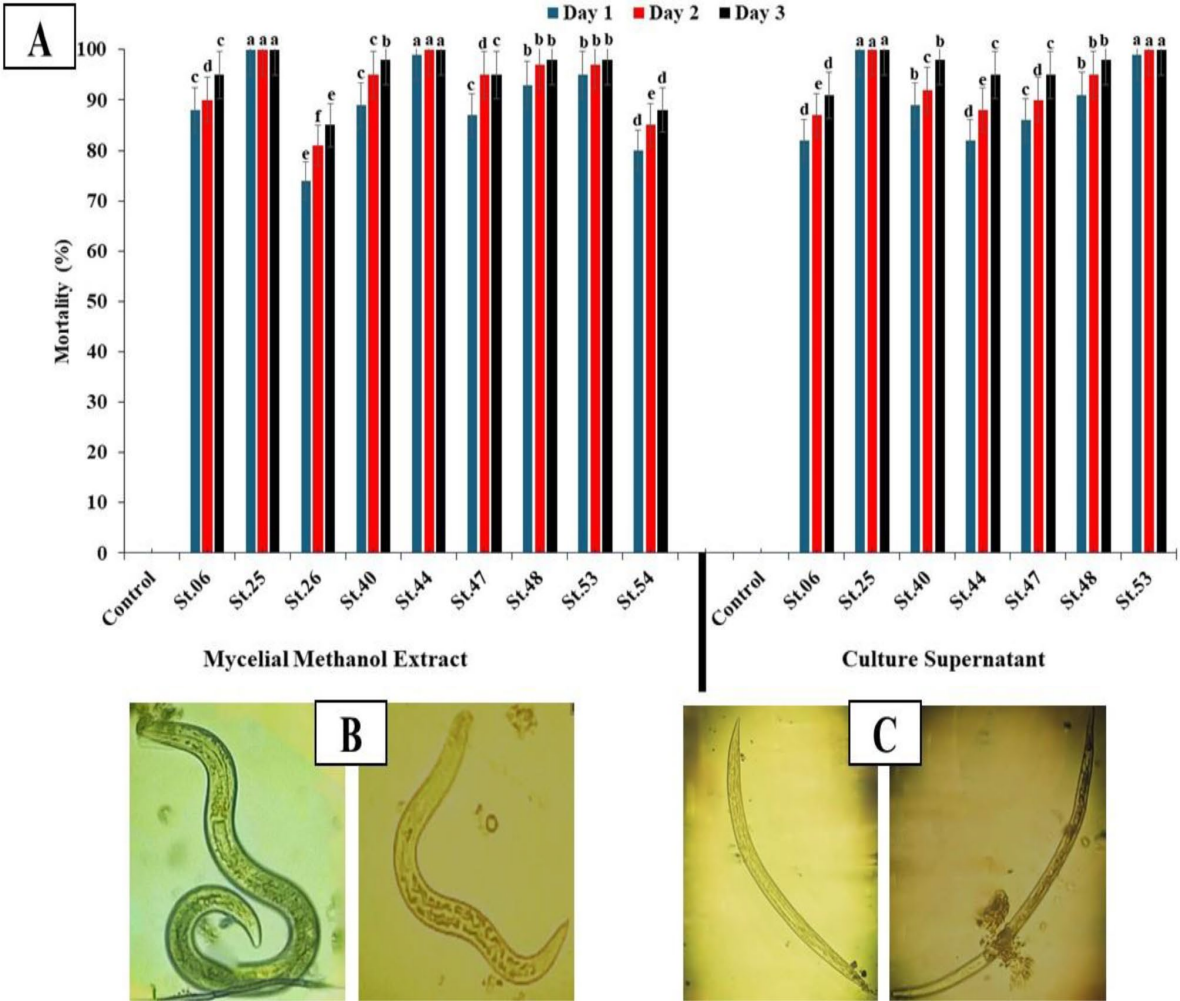
**(A)** The actinobacterial isolates’ nematicidal potential against second-stage juveniles of *M. incognita* expressed as mortality (%) during three consequence days at 25 °C. **(B)** Morphological observation of the active nematodes. **(C)** The dead nematodes with straight form and immobility, post-stimulation using a fine needle. *Values are the mean of 3 replicates. Averages followed by the same letter(s) are not significantly (*P* ≤ 0.05) different according to Duncan’s Multiple Range Test

Data documented in Fig. (1), shows only isolates with mortality ≥ 74% as the lower mortality isolates were neglected. The isolates St.25, St.44, and St.53 revealed the highest significant nematicidal potentiality against *Meloidogyne* J_2_s (Fig. 1). According to the mortality results, the mycelial-methanol extract and the supernatant extract of isolate St.25 exhibited the maximum mortality of 100% from day one followed by the mycelial-methanol extract of the isolate St.44 and the supernatant extract of the isolate St.53 with 99% mortality. 100% mortality was observed on the second and third days in both extracts.

### Isolates’ efficiency in controlling *M. incognita* on tomato

**Fig. 2:**
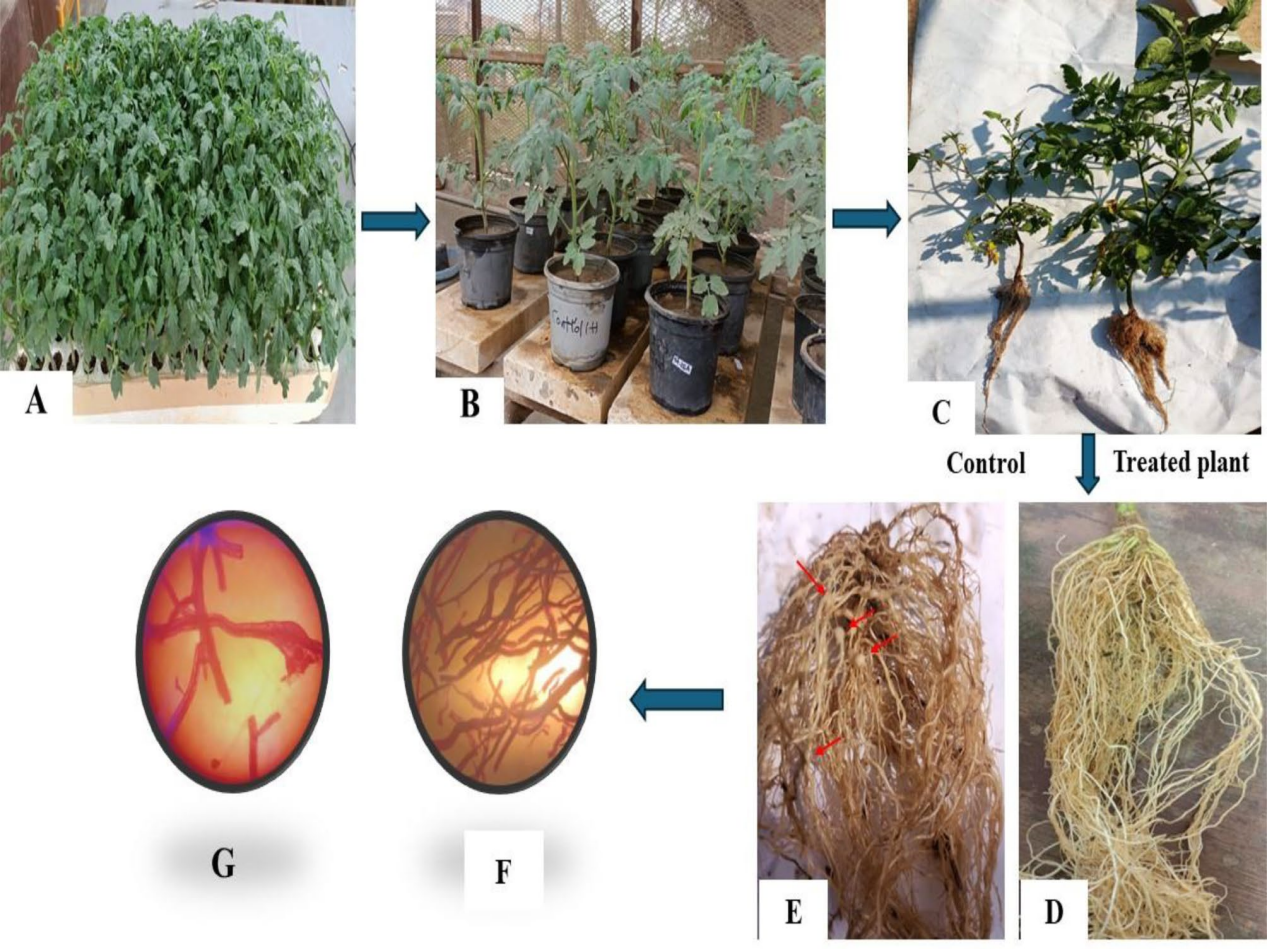
Effect of the selected isolates on controlling *M. incognita* on tomato plants in a pot experiment. **(A)** Tomato seedlings with true leaf stage. **(B)** Tomato plants infested with *M. incognita* and treated with the selected isolates. **(C)** Control tomato plant infected with nematode only, and tomato plant infected with nematode and treated with methanol-extracted mycelium of the isolate St.53. **(D)** The effect of different treatments on reducing the number of goals on roots. **(E)** The arrows indicate goals on tomato-infected roots. **(F & G)** Plant roots were stained using lactophenol dye under a stereomicroscope

The nematicidal effectiveness of the four selected actinobacterial isolates (St.25, St.44, St.48, and St.53) against *M. incognita* infecting tomatoes were evaluated in a greenhouse condition by counting the number of nematode juveniles (J_2_s) {females, galls, and egg masses} in the soil and roots, and then the results were compared with the control (Tables 1 and Fig. 2).

The four tested isolates demonstrated a suppressive effect on *M. incognita*, as evidenced by the significant reductions in the number of the studied nematode’s reproductive criteria and galls, as well as the average nematode reproduction rate, at different extents.The methanol-extracted mycelium of isolate St.53 outperformed other tested isolates in reducing all nematode parameters. It demonstrated the highest nematode reduction of 75.69%, followed by the culture suspension of 75.38%. Additionally, the culture suspension of isolate St.53 exhibited the lowest reproduction rate (Rr) of 0.80, followed by methanol-extracted mycelium of 0.84.

**Table 1:** Effect of the selected isolates on the mortality and reproduction of *M. incognita* infecting tomato plants

Treatments		Nematodes reproductive parameters and galls (No./plant/pot)												
		J_2_s in soil	Red. %	J_2_s in roots	Red. %	Females	Red. %	Egg-masses	Red. %	Galls	Red. %	Average of total reduction %	Reproduction rate (Rr)	
Control without Nematode		0^h^	100	0 g	100	0 h	100	0i	100	0j	100	100.00	0.00	
Nematode only		3150^a^	0	930^a^	0	217a	0	182a	0	194a	0	0.00	4.08	
Culture suspension	St.25	1623^b^	48	417^e^	55	139^c^	36	115^d^	37	126^cd^	35	42.30	2.04	
	St.44	1597^b^	49	457^cde^	51	147^bc^	32	123^c^	33	133^c^	32	39.37	2.05	
	St.48	973^ef^	69	517^c^	44	107^e^	51	76^f^	58	85^g^	56	55.70	1.49	
	St.53	593^g^	81	203^f^	78	63^g^	71	48^h^	74	52^i^	73	75.38	0.80	
Culture Supernatant	St.25	1257^cd^	60	433^e^	53	143^c^	34	126^c^	31	128^cd^	34	42.51	1.69	
	St.44	1207^de^	62	597^b^	36	142^c^	35	115^d^	37	125^d^	36	40.93	1.80	
	St.48	1480^bc^	53	517^c^	44	126^d^	42	109^de^	40	115^e^	41	44.01	2.00	
	St.53	710^fg^	77	193^f^	79	77^f^	64	65^g^	64	68^h^	65	70.02	0.90	
Mycelial Methanol Extract	St.25	1357^bcd^	57	440^de^	53	156^b^	28	135^b^	26	145^b^	25	37.76	1.80	
	St.44	1443^bcd^	54	580^b^	38	147^bc^	32	124^c^	32	128^cd^	34	37.89	2.02	
	St.48	1210^cde^	62	500^cd^	46	110^e^	49	103^e^	43	96^f^	51	50.21	1.71	
	St.53	650^g^	79	190^f^	80	63^g^	71	47^h^	74	50^i^	74	75.69	0.84	
LSD 5%		270.375	--	61.884	--	9.397	--	6.295	--	7.285	--	--	--	

Averages followed by the identical letter (s) within each column are not significantly (*P* ≤ 0.05) different according to the LSD 5% Test

The isolate St.53 demonstrated superiority in increasing all growth parameters of tomatoes infected by *M. incognita* compared to other tested isolates. Its culture suspension showed the highest significant increase in shoot length (61 cm), shoot fresh weight (21.34 g), shoot dry weight (3.052 g), root length (26.33 cm), number of leaves (13), number of flowers (5), and the average total percentage increase in all measured parameters of 65.56% (Tables 2 and Fig. 2).

**Table 2:** Effect of the selected isolates on the growth parameters of tomatoes infected by *M. incognita*

Treatments		Plant growth parameters/plant													
		Shoot length (cm)	% Inc.	Shoot fresh weight (g)	% Inc.	Shoot dry weight (g)	% Inc.	Root length (cm)	% Inc.	No. of leaves	% Inc.	No. of flowers	% Inc.	The average of total increase percentage	
Control		54.33^bcd^	66.33	16.11^de^	20.16	2.220^f^	21.86	22.67^bc^	51.11	11^ab^	13.33	4^ab^	33.33	34.35	
Nematode only		32.67^g^	0.00	13.41^f^	0.00	1.822^g^	0.00	15.00^h^	0.00	10^b^	0.00	3^bc^	0.00	0.00	
Culture suspension	St.25	41.00^ef^	25.51	16.54^de^	23.32	2.642^cd^	45.00	23.00^bc^	53.33	12^ab^	20.00	5^a^	55.56	37.12	
	St.44	51.00^cd^	56.12	17.93^bcd^	33.73	2.815^bc^	54.52	21.00^cd^	40.00	12^ab^	16.67	5^a^	77.78	46.47	
	St.48	51.67^cd^	58.16	17.31^cd^	29.08	2.752^bc^	51.04	24.00^b^	60.00	11^ab^	10.00	4^ab^	33.33	40.27	
	St.53	61.00^a^	86.73	21.34^a^	59.13	3.052^a^	67.49	26.33^a^	75.56	13^a^	26.67	5^a^	77.78	65.56	
Supernatant	St.25	44.33^e^	35.71	15.75^def^	17.42	2.618c^de^	43.67	19.00^def^	26.67	11^ab^	13.33	5^a^	66.67	33.91	
	St.44	37.00^fg^	13.27	15.85^def^	18.20	2.426^ef^	33.13	16.67^gh^	11.11	12^ab^	16.67	4^ab^	33.33	20.95	
	St.48	50.67^cd^	55.10	17.50^bcd^	30.47	2.632^cde^	44.46	23.33^b^	55.56	10^b^	0.00	3^bc^	11.11	32.78	
	St.53	58.67^ab^	79.59	19.93^ab^	48.60	2.899^ab^	59.09	24.33^ab^	62.22	12^ab^	16.67	5^a^	55.56	53.62	
Mycelial Methanol Extract	St.25	50.33^d^	54.08	14.54^ef^	8.45	2.416^ef^	32.58	20.33^de^	35.56	10^b^	0.00	4^ab^	44.44	29.74	
	St.44	44.67^e^	36.73	16.05^def^	19.69	2.525^de^	38.60	18.33^efg^	22.22	10^b^	0.00	3b^c^	0.00	19.54	
	St.48	37.33^f^	14.29	17.72^bcd^	32.14	2.661^cd^	46.03	18.00f^g^	20.00	10^b^	0.00	3^bc^	11.11	21.15	
	St.53	55.00^bc^	68.37	19.76^abc^	47.33	2.946^ab^	61.71	23.00^bc^	53.33	13^a^	30.00	5^a^	77.78	56.42	
LSD 5%		4.402	--	2.603	--	0.216	--	2.204	--	2.032	--	1.878	--	--	

Averages followed by the identical letter (s) within each column are not significantly (*P* ≤ 0.05) different according to the LSD 5% Test

### The physiological and biochemical properties of the isolate St.53

**Fig. 3:**
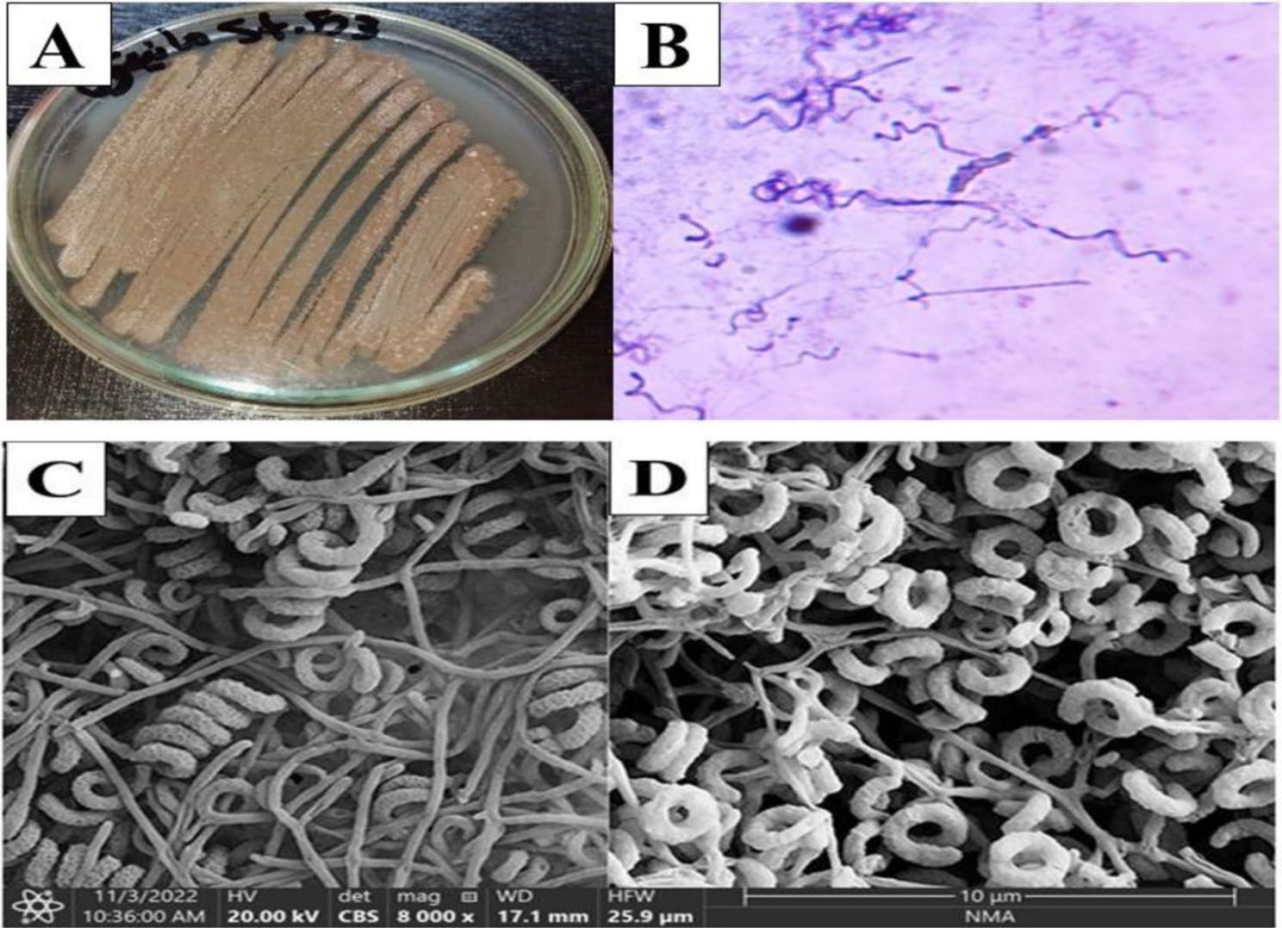
Phenotypic characteristics of the isolate St.53 **(A)** cultural characteristics on starch nitrite agar. **(B)** Microphotograph of spore chain morphology (1000x). **(C)** Morphological characteristics of aerial mycelium by SEM. **(D)** Morphological characteristics of spores by SEM

The phenotypic characteristics of the isolate St.53 are illustrated in Tables 3and Fig. 3. The isolate St.53 showed a powdery, brownish gray mixed with white aerial mycelium, and dark brown vegetative mycelium in addition to producing light brown, soluble pigments. In addition, the isolate St.53 revealed the capability to produce melanoid pigment on tyrosine agar, and peptone-yeast extract iron agar media. This strain was distinguished for its ability to produce spherical to oval spores on an open spiral sporophore, grow on Czapek’s agar medium, tolerate sodium chloride from 4 to 7% NaCl, and sensitive to 50 µg/mL streptomycin.

It revealed its capability to use D-glucose, D-xylose, L-arabinose, L-rhamnose, D-fructose, galactose, raffinose, D-mannitol, inositol, maltose, and sucrose as carbon sources. Additionally, it showed the capability to produce amylase, lipase, gelatinase, catalase, and oxidase but lacked the capability to produce cellulase (Table 3). Accordingly, isolate St.53 was selected for phylogenetic analysis based on 16 S rRNA gene sequencing and assessing its capability to produce abamectin as a secondary metabolite since it was the only isolate that exhibited resemblance to *S. avermitilis*.

**Table 3:** Phenotypic characteristics of the isolate St.53

Phenotypic characteristics	Tests	St.53
Cultural characteristics	Color of aerial mycelium	Brownish grey mixed with white
	Color of substrate mycelium	Dark brown
	Diffusible pigments	Light brown
Morphological characteristics	Spore surface ornamentation	Open spiral sporophores carry oval spores
	Spore chain morphology	Spiral
Physiological characteristics	Melanoid pigment produced on tyrosine agar	+Ve
	Melanoid pigment produced on peptone-yeast extract iron agar media	+Ve
	Growth on Czapek’s medium	+Ve
	Sodium chloride tolerance	Tolerant to Sodium chloride from 4 to 7%
	Sensitivity to Streptomycin (50 µg/mL)	Sensitive
Biochemical tests	Amylase	+Ve
	Lipase	+Ve
	Gelatinase	+Ve
	Cellulase	-Ve
	Catalase	+Ve
	Oxidase	+Ve
Growth in carbon sources	Control (no carbon source)	-
	D-Glucose	+++
	D-Xylose	+++
	L-Arabinose	+++
	L-Rhamnose	+++
	D-Fructose	+++
	Galactose	+++
	Raffinose	+++
	D-Mannitol	+++
	Inositol	+++
	Maltose	+++
	Sucrose	+++

### Identification of abamectin produced by the isolate St.53 using LC-MS

**Fig. 4:**
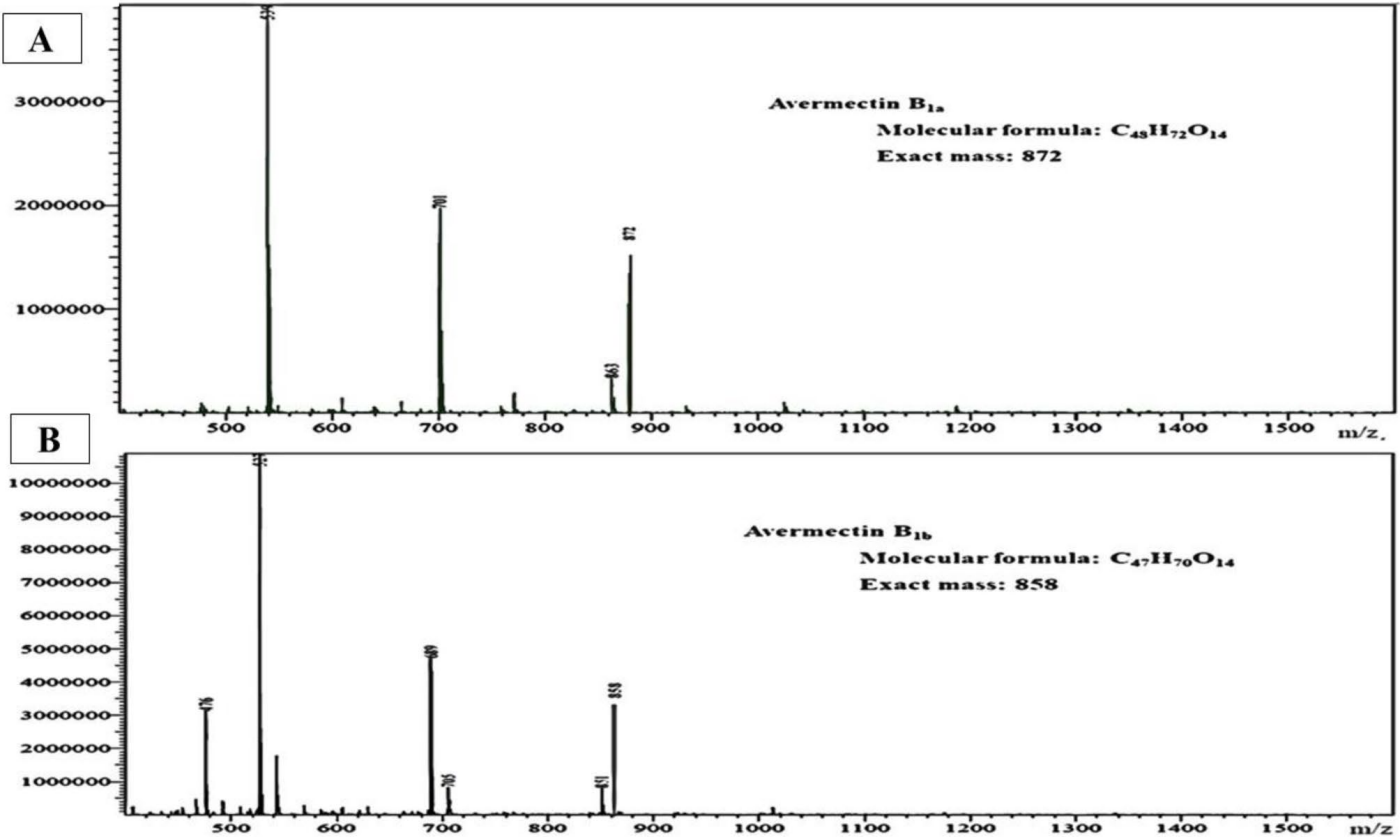
**(A)** Liquid chromatography-mass spectrometry (LC-MS) of AVE B1a (Molecular formula: C_48_H_72_O_14_ and Exact mass: 872) extracted from *Streptomyces* isolate St.53 - methanol extracts. **(B)** Liquid chromatography-mass spectrometry (LC-MS) of AVE B1b (Molecular formula: C_47_H_70_O_14_ and Exact mass: 858) extracted from *Streptomyces* isolate St.53- methanol extracts

Abamectin produced by the isolate St.53 was identified by LC-MS and found abamectin B1a (molecular mass 872) and abamectin B1b (molecular mass 858) where their spectra are presented in Fig. (4 A, B) respectively.

### Quantification of the produced abamectin B1b and B1a using HPLC

**Fig. 5:**
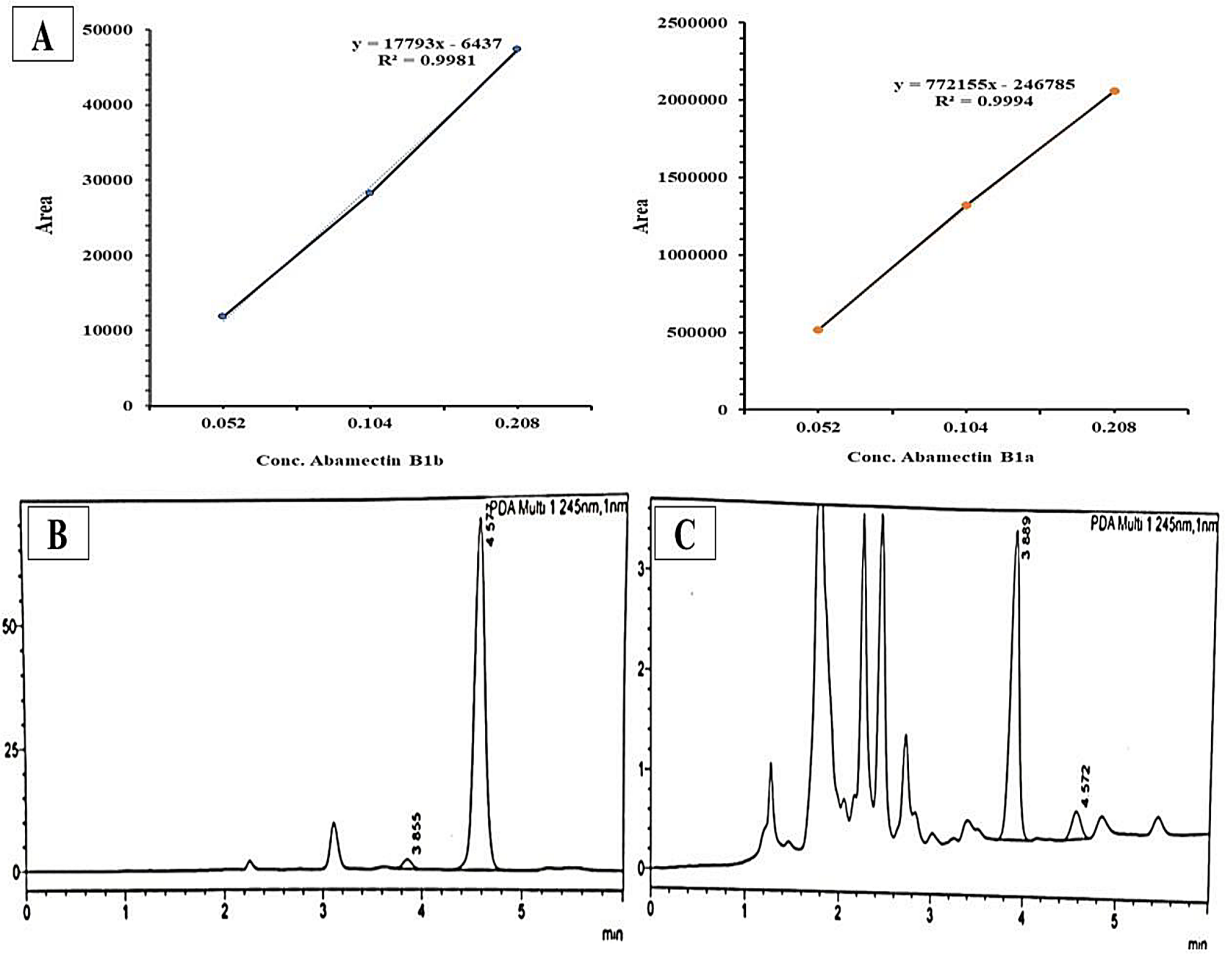
**(A)** The concentrations of standard abamectin B1b and B1a (mg/mL), represented as standard curves. **(B)** Standards of abamectin B1b (Rt 3.855 min) and B1a (Rt 4.577 min) by HPLC analysis, **(C)** Quantification of abamectin B1b and B1a produced by the cell extract of *Streptomyces* isolate St.53 using HPLC analysis

The concentration of abamectin’s components (B1a, B1b) was determined quantitatively by HPLC. The standard curve of abamectin (B1a and B1b) was constructed depending on the peak area of each standard concentration (Fig. 5A). Considering the HPLC profiles, The standards of abamectin B1b and B1a appeared at retention times 3.855 and 4.572 min, respectively (Fig. 5B).

The peak area of the mycelium extracted sample was1975 for B1a and 19,465 for B1b (Fig. 5C) corresponding to concentrations 0.0855 mg/mL and 0.0002 mg/mL respectively (Table 4). The concentration of abamectin B1a and B1b in the mycelium-extracted sample was indicated by the standard curves for abamectin B1a and B1b, which gave the equations Y = 772155x – 246,785; R² = 0.9994; and Y = 17793x – 6437; R² = 0.9981, respectively. The yield of abamectin (B1a and B1b) was determined by calculating the dilution of the used sample generated by *Streptomyces* isolate St.53 after 10 days of incubation, revealing a yield of 3.238 g/L. (Fig. 5 and Table 5). Each component could not be quantified separately by the HPLC method because of the low concentration of A B1a.

**Table 4:** Abamectin B1b and B1a quantification using HPLC analysis

Samples	Peak	Ret. Time	Area	Height	Concentration	Unit	Name
Standard abamectin	1	3.855	11,799	1913	0.052	mg/mL	Abamectin B1b
	2	4.577	514,270	70,811	0.052	mg/mL	Abamectin B1a
	Total		526,070	72,725			
*Streptomyces* isolate - St.53 (cell extract)	1	3.888	19,412	3100	0.0855	mg/mL	Abamectin B1b
	2	4.572	2009	283	0.0002	mg/mL	Abamectin B1a
	Total		21,421	3383			

### Molecular Identification of Isolate St.53

The isolate St.53 was identified using 16SrRNA gene sequence analysis, and the neighbor-joining technique was utilized to assemble the phylogenetic tree using the distance values.

**Fig. 6:**
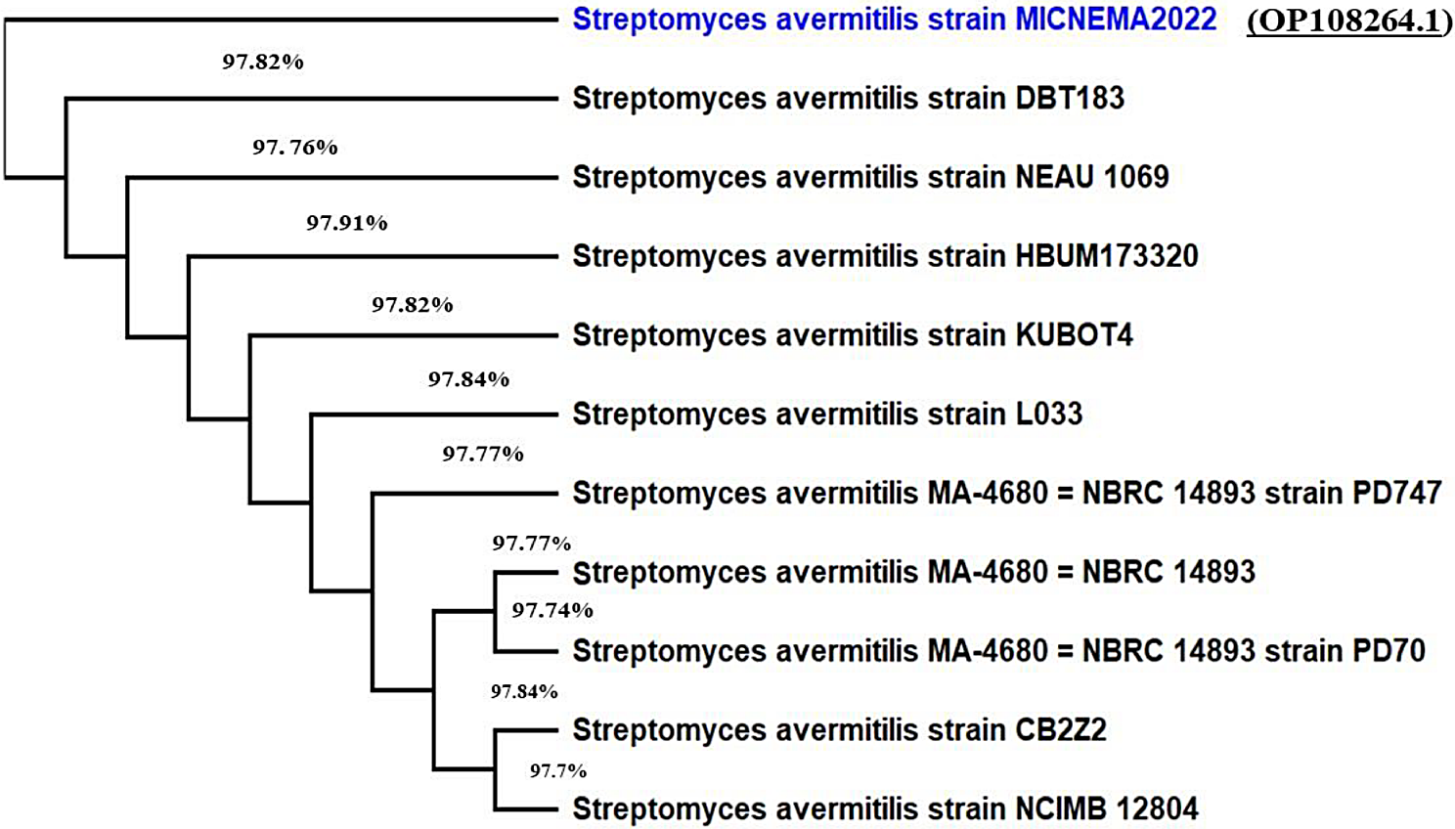
Phylogenetic neighbor-joining tree based on the 16 S rRNA gene sequence of St.53 showing the relationship to the closest phylogenetic relative *Streptomyces avermitilis*

The 16 S rRNA gene sequence closely resembled that of *S. avermitilis* (NCBI). This sequence was submitted to the GenBank database (http://www.ncbi.nlm.nih.gov/GenBank) as *S. avermitilis* St.53 with accession number No. OP108264.1, and it revealed high similarity to all members of *S. avermitilis* present on the GenBank database. The findings revealed 97% sequence similarity to 10 *S. avermitilis* strains. MEGA X program, phylogenetic analysis was performed using the neighbor-joining algorithm to reveal the similarity between the isolate and its nearest phylogenetic neighbors (Fig. 6). Accordingly, based on morphological, cultural, and physiological characteristics as well as 16 S rRNA sequence, the obtained strain was identified as *S. avermitilis* St.53.

### Molecular modeling of the binding of abamectin (Avermectin B1a and B1b) with glutamate-gated chloride channel (GluCL)

**Fig. 7:**
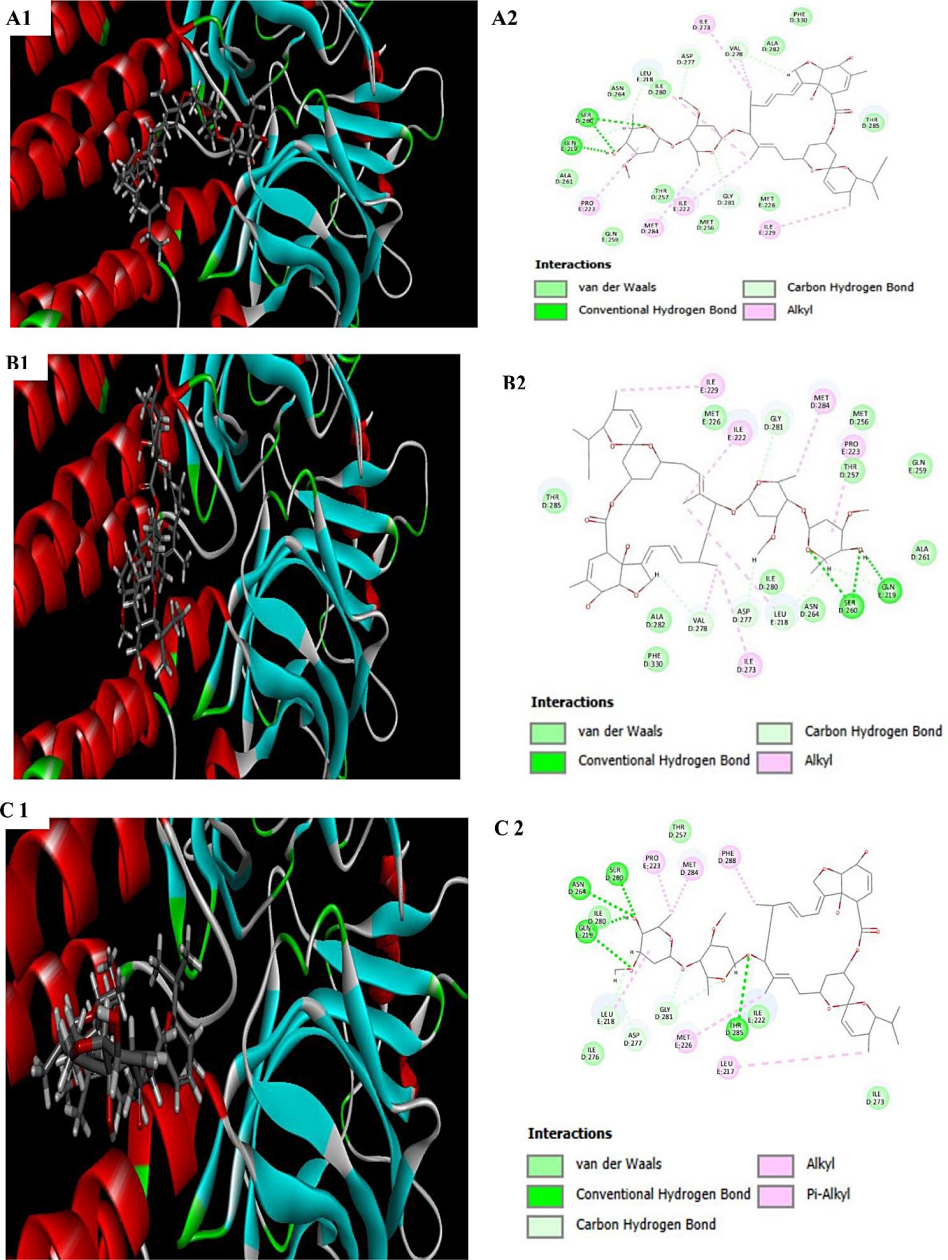
The molecular dockings of abamectin B1a and B1b with GluCL. It shows the binding orientations of ligands **(A1)** Ivermectin, **(B1)** Abamectin B1a, and **(C1)** Abamectin B1b, with the active sites of GluCL. The protein is represented by ribbons, and backbone hydrogen bonds are indicated by dotted yellow lines. Interactions between the respective ligands and surrounding amino acid residues are shown in **(A2)**,** (B2)**, and **(C2)**, along with sidechain hydrogen bonds indicated by dotted pink arrows

Ivermectin exhibited strong binding with the GluCL receptor (binding energy: −72.35 kcal/mol) in our docking simulation (Fig. 7A1). Interactions were stabilized by strong backbone hydrogen bonding with leucine 218 of the GluCL receptor and by a weaker side chain hydrogen bonding with glutamine 219. Abamectin B1a revealed a glide score of − 90.76 kcal/mol, without any side chain or backbone hydrogen bonding (Fig. 7B1). Abamectin B1a scored − 92.85 kcal/mol, also lacking side chain or backbone hydrogen bonding (Fig. 7C1). As illustrated in Fig. 5, all the compounds displayed the potential to interact with the essential amino acid residues located in the active sites of the targeted proteins via a stable set of hydrophilic and hydrophobic interactions. Additionally, these compounds were also capable of binding to other amino acid residues.

Our findings revealed that abamectin B1b was the most effective nematicide in terms of binding to the GluCL receptors in C. elegans. It revealed the ability to create a network of both hydrophilic and hydrophobic interactions within the binding pockets of the targeted proteins, leading to high docking scores (Fig. 7 and Table 5). Abamectin B1a exhibited significant binding affinity scores towards the targeted proteins, similar to B1b and ivermectin. This binding is crucial for maintaining the open pore structure of the GluCL complex. By strongly binding with the active sites of the GluCL receptor, the abamectin B1a and B1b compounds keep the ion channel open and allowing fluid intake.

**Table 5:** Binding interactions and scores of abamectin B1a and B1b, as well as ivermectin in the glutamate-gated chloride channel receptor active site of C. Elegans

No.	Ligand compound	Binding Score (kcal/mol)	Bond interactions		
			Bond type	Length (Aº)	Residues
1	Ivermectin	-72.35	Van der Waals	1.09	ALAD:261
				1.08	ALAD:282
				1.53	ASND:264
				1.07	GLNE:259
				1.09	ILED:280
				1.51	METE:226
				1.09	METD:256
				1.53	PHED:330
				1.09	THRD:256
				0.95	THRD:285
			Conventional hydrogen bond	1.33	GLNE:219
				1.52	SERD:260
			Carbon hydrogen bond	1.25	ASPD:277
				1.22	GLYD:281
				1.08	LEDE:218
				1.53	VALD:278
			Alkyl	1.09	ILEE:222
				1.08	ILEE:229
				1.09	ILED:273
				1.80	METD:284
				1.50	PROE:223
2	Abamectin B1a	-90.76	Van der Waals	1.08	ALAD:261
				1.09	ALAD:282
				1.53	ASND:264
				1.09	ILED:280
				1.08	METD:256
				1.51	METE:226
				1.49	PHED:330
				0.85	THRD:257
				1.08	THRD:285
			Conventional hydrogen bond	1.52	SERD:260
				1.07	GLNE:219
			Carbon hydrogen bond	1.24	ASPD:277
				1.22	GLYD:281
				1.08	LEUE:218
				1.09	VALD:278
			Alkyl	1.09	ILED:273
				1.08	ILEE:222
				1.09	ILEE:229
				1.48	PROE:223
3	Abamectin B1b	-92.85	Van der Waals	1.09	ILEE:222
				1.08	ILED:273
				1.09	ILED:276
				1.07	ILED:280
			Conventional hydrogen bond	1.53	ASND:264
				1.33	GLNE:219
				1.52	SERD:260
				0.95	THRD:285
				1.09	THRD:257
			Carbon hydrogen bond	1.24	ASPD:277
				1.22	GLYD:281
				1.08	LEUE:218
			Alkyl	1.08	METD:284
				1.38	PHED:288
				1.48	PROE:223
			Pi-Alkyl	1.09	LEUE:217
				1.80	METE:226

## Discussions

Abamectin is a highly effective bionematicide for controlling plant parasitic nematodes, particularly root-knot nematodes (RKNs). Its nematicidal efficacy has been widely documented and recognized [44–46]. The main emphasis in the present investigation was the isolation and selection of the best *Streptomyces* spp., which produces abamectin as an anthelmintic agent.

Geographical location and soil type had a substantial impact on the diversity of the actinobacterial isolates in the soil [23, 47, 48].

The color of the substrate and aerial mycelium, the formation of soluble pigments, and the characteristics of the spores were used to characterize the morphology of the actinobacterial species, which revealed typical characteristics of the genus *Streptomyces* [23, 49–53]. Therefore, these isolates were preliminarily identified as *Streptomyces* sp. according to their morphological differentiation [15, 54, 55]. The presence of color variations in the selected isolates is in line with the previously published data for *Streptomyces* strains [49]. The morphological examination of the selected isolates verified them as *Streptomyces* sp. [15, 21, 49, 51, 53].

The actinobacterial isolates’ nematicidal potential was evaluated on *M. incognita* second-stage juveniles (J_2_s) as described above using two types of actinobacterial extracts [56, 57]. The outcomes of this experiment revealed mortality ranged from 5 to 100%, which is consistent with those stated earlier [58] claiming that *S. avermitilis* can demonstrate nematicidal qualities against *M. incognita* with variable degrees.

Furthermore, it is reported that avermectin formed by *Streptomyces* sp. significantly decreased the hatching of juveniles of *M. incognita* during the first day and was completely suppressed on the second day [58]. In the same consequence, the present findings are also supported by previous reports [13, 59–61] stating that avermectin solution expressed high capability in inhibiting the egg-hatching of *M. arenaria.* In addition, the outcomes of the present investigation are in conformity with reports mentioning that culture filtrates of different *Streptomyces* isolates proved their capability to induce the mortality of second-stage juveniles of *M. incognita* [58].

The proficiency of the selected isolates in controlling *M. incognita* on tomato in a pot experiment revealed the suppressive effect of the tested isolates on *M. incognita*, which aligns with the findings reported by [14, 17, 58] on diverse vegetable crops. Correspondingly, these results are in conformity with those stated by [14, 62].

The phenotypic characteristics of actinobacterial isolate St.53 are consistent with the previous results [12, 53] and Bergey’s Manual of Systematic Bacteriology [27].

*Streptomyces avermitilis* is highly efficient in producing avermectins as secondary metabolites [15]. The avermectins consist of eight fractions (A1a, A2a, A2b, A1b, B1a, B2a, B1b, and B2b), among which abamectin (a mixture of B1a and B1b) is the commonest constituent and has the strongest activity against parasites [55].

HPLC and LC-MS analysis of methanol-extracted mycelium confirmed the capability of the isolate St.53 to produce abamectin. Abamectin production by this isolate was found to be within the range reported previously [15, 39], but higher (10.15 mg/L by *Streptomyces avermitilis*) [49] and lower (12.8118 mg/L to 17.7798 mg/L of B1b) [23] than values found in other reports.

The comparison of 16 S rRNA gene sequences is a powerful method for the evaluation of phylogenetic and evolutionary relationships between bacterial strains [15]. The 16 S rRNA gene is utilized in molecular identification due to its stability and comparability for determining phylogenetic relationships between microorganisms [15, 63]. The 16 S rRNA sequence analysis of *Streptomyces* sp. was contrasted with that of other *Streptomyces* strains. Considering the gathered data, and the comparative studies of this isolate with the closest members of the *Streptomyces* species, it revealed a close relation to the type strains of *S. avermitilis* with accession number OP108264.1. [40, 53]. Based on morphological, cultural, and physiological characteristics as well as 16 S rRNA sequence, the obtained strain was identified as *S. avermitilis* St.53.

Abamectin (Avermectin B1a and B1b) is a nematicidal compound that is used to kill parasitic nematodes. It works by binding to the GluCL receptor and opening the ion channel [64, 65], which causes a rapid influx of chloride ions into cells. This leads to membrane hyperpolarization and subsequent paralysis. Molecular docking modeling was conducted to explore the binding interactions of abamectin B1a and B1b, isolated from *Streptomyces avermitilis* MICNEMA2022 strain.

## Conclusion

The current investigation has successfully isolated *S. avermitilis* MICNEMA2022 strain. It demonstrated a suppressive effect against the *M. incognita* infecting tomato plants, as evidenced by the significant reductions in the number of the studied nematode’s reproductive criteria and galls, as well as the average nematode reproduction rate, at different extents. Its culture suspension demonstrated superiority in increasing all growth parameters of tomatoes infected by *M. incognita*. High-performance liquid Chromatography analysis of the mycelial extract showed the production of abamectin (B1a and B1b) illustrated by a specific peak. The LC-MS confirmed the presence of abamectin’s derivatives in the form of AVE B1a, and AVE B1b, which was the target of this investigation.

## Electronic supplementary material

Below is the link to the electronic supplementary material.

Supplementary Material 1

Supplementary Material 2

Supplementary Material 3

## Data Availability

The data sets used and/or analyzed during the current study are available from the corresponding author upon reasonable request. Streptomyces avermitilis MICNEMA2022 with accession number (OP108264.1) was deposited in GenBank at (https://www.ncbi.nlm.nih.gov/nuccore/OP108264).
