# Supplementary material for: Streptomyces avermitilis MICNEMA2022: a new biorational strain for producing abamectin as an integrated nematode management agent
Source: BMC Microbiol. 2024 Sep 7;24:329. doi: 10.1186/s12866-024-03466-3 (PMC11380338; doi:10.1186/s12866-024-03466-3)
Supplement: Supplementary file 1 — Supplementary Material 1 [file 12866_2024_3466_MOESM1_ESM.docx]

**Table (S1):** The frequency of actinobacteria isolation throughout the nine studied sites.

| **Collection site** | **Type of soil** | **Number of collected isolates** | **Incidence of isolates (%)** |
| --- | --- | --- | --- |
| Monofiya | Clay soil | 5 | 8.4 |
| Sohag | Clay soil | 6 | 10 |
| Giza | Clay soil | 1 | 1.6 |
| Beni Suef | Clay soil | 7 | 11.7 |
| Beheira | Sandy soil | 3 | 5 |
| Qalioubiya | Clay soil | 10 | 16.6 |
| Ismailia | Sandy loamy soil | 15 | 25 |
| Fayoum | Sandy loamy soil | 7 | 11.7 |
| Gharbiya | Clay soil | 6 | 10 |
| Total | - | 60 | 100 |

**Table (S2):** Grouping of the actinobacterial isolates based on their mycelium color

| **Color of aerial mycelium** | **Total isolates** | **%** |
| --- | --- | --- |
| Grey | 30 | 50 |
| White | 12 | 20 |
| Green | 11 | 18 |
| Brown | 5 | 8 |
| Red | 2 | 4 |
| Total | 60 | 100 |
