# Supplementary material for: Streptomyces avermitilis MICNEMA2022: a new biorational strain for producing abamectin as an integrated nematode management agent
Source: BMC Microbiol. 2024 Sep 7;24:329. doi: 10.1186/s12866-024-03466-3 (PMC11380338; doi:10.1186/s12866-024-03466-3)

(S5): LC-MS Identification of abamectin produced by the isolate St.53 using LC-MS

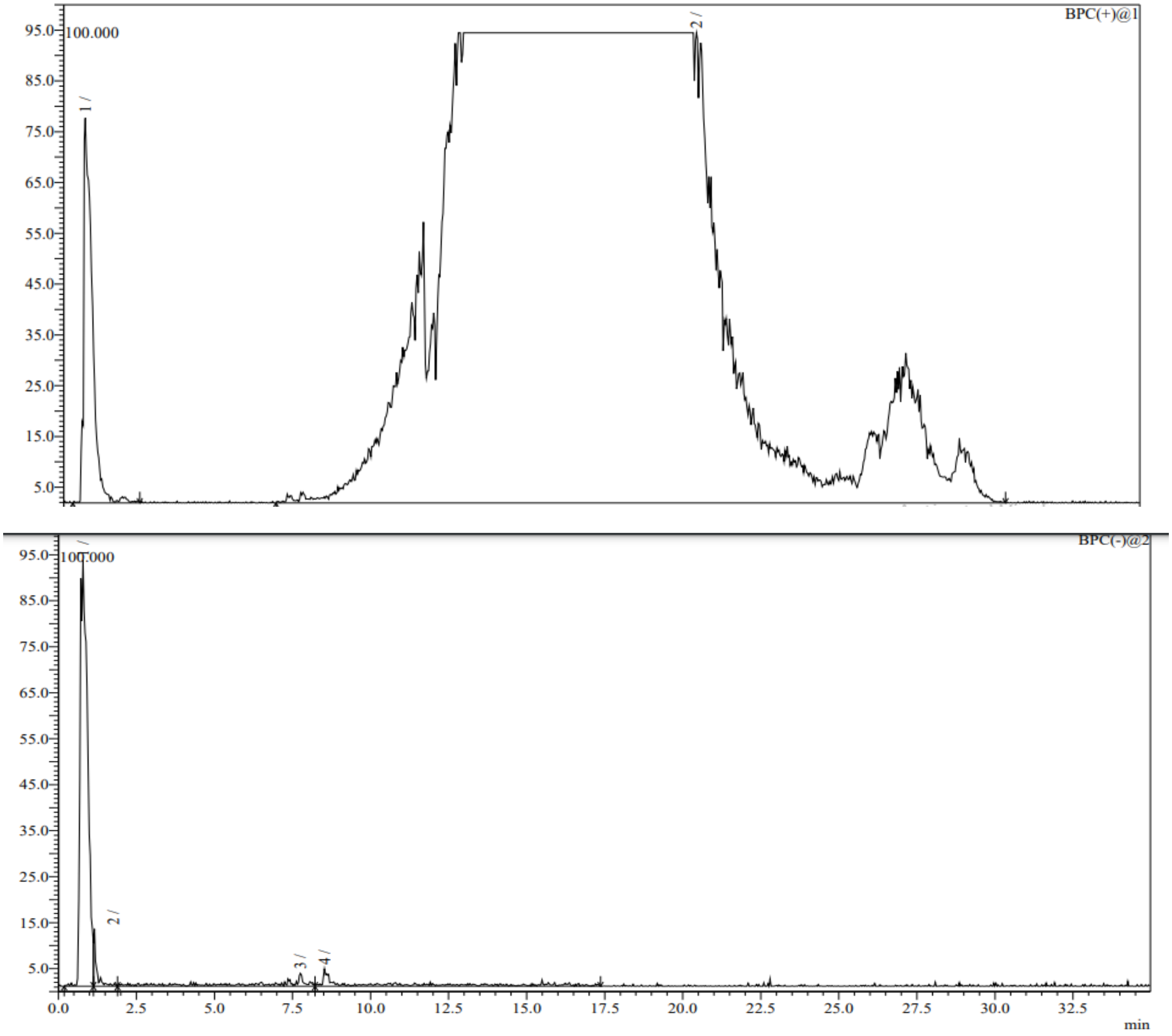

MASS Peak Table BPC

Peak#	ompound	Ret. Time	Event#	Base Peak m/z	m/z	Area	A/H	Area%
1		0.70	1-1	527.10	BPC	317146226	19.444	2.432
2		20.57	1-1	413.25	BPC	###########	638.706	97.568
3		0.79	1-2	539.15	BPC	74305231	16.671	76.980
4		1.15	1-2	407.05	BPC	3868558	6.422	4.008
5		7.76	1-2	581.30	BPC	7966415	58.257	8.253
6		8.53	1-2	593.20	BPC	10385144	56.369	10.759
Total						###########		200.000

MS Spectrum

1?----Line#:1 R.Time:----(Scan#:----)
MassPeaks:10
Spectrum Mode:Averaged 0.64-0.70(39-43) Base Peak:527(10895253)
BG Mode:Calc Segment 1 - Event 1

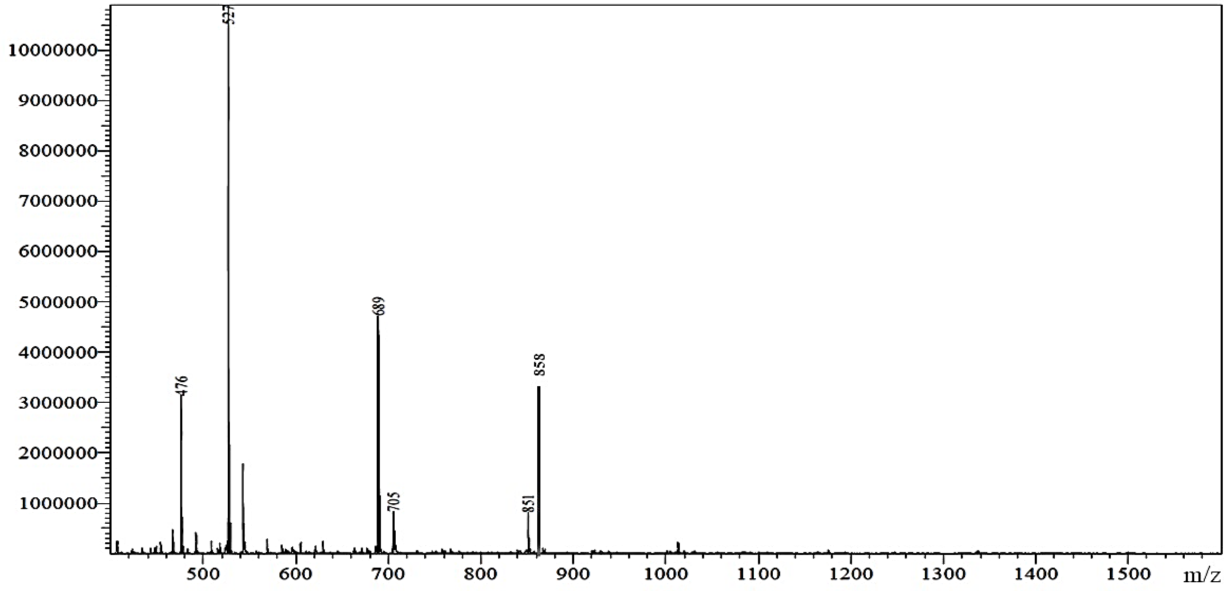

2?----Line#:2 R.Time:----(Scan#:----)
MassPeaks:7
Spectrum Mode:Averaged 20.54-20.60(1227-1231) Base Peak:413(19109913)
BG Mode:Calc Segment 1 - Event 1

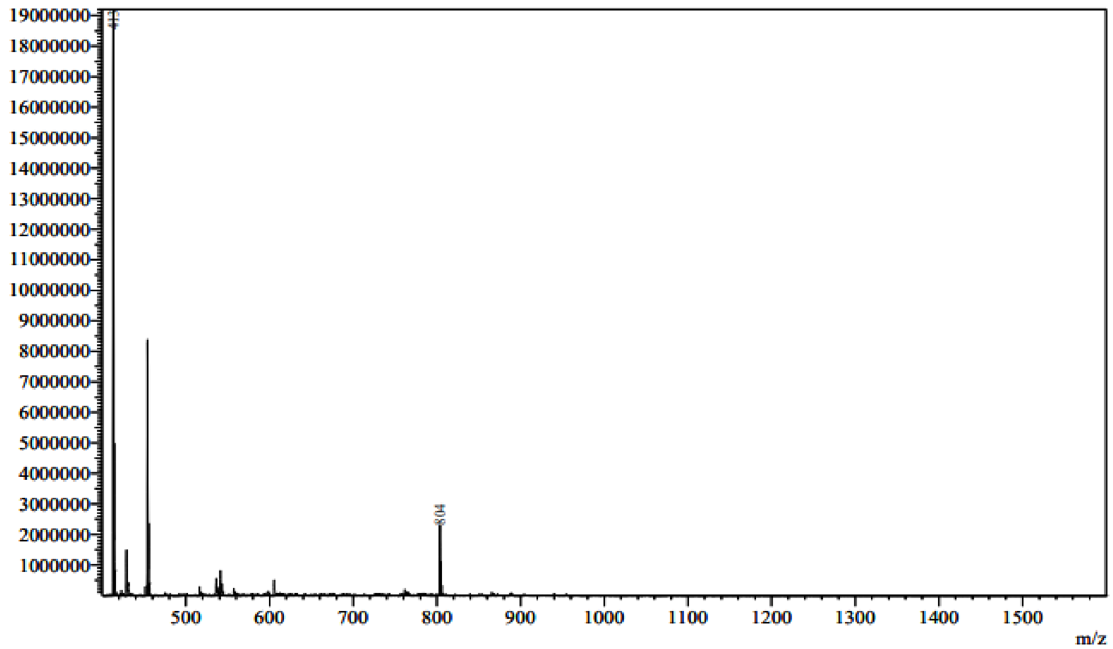

3?----Line#:3 R.Time:----(Scan#:----)
MassPeaks:8
Spectrum Mode:Averaged 0.75-0.82(46-50) Base Peak:539(3888938)
BG Mode:Calc Segment 1 - Event 2

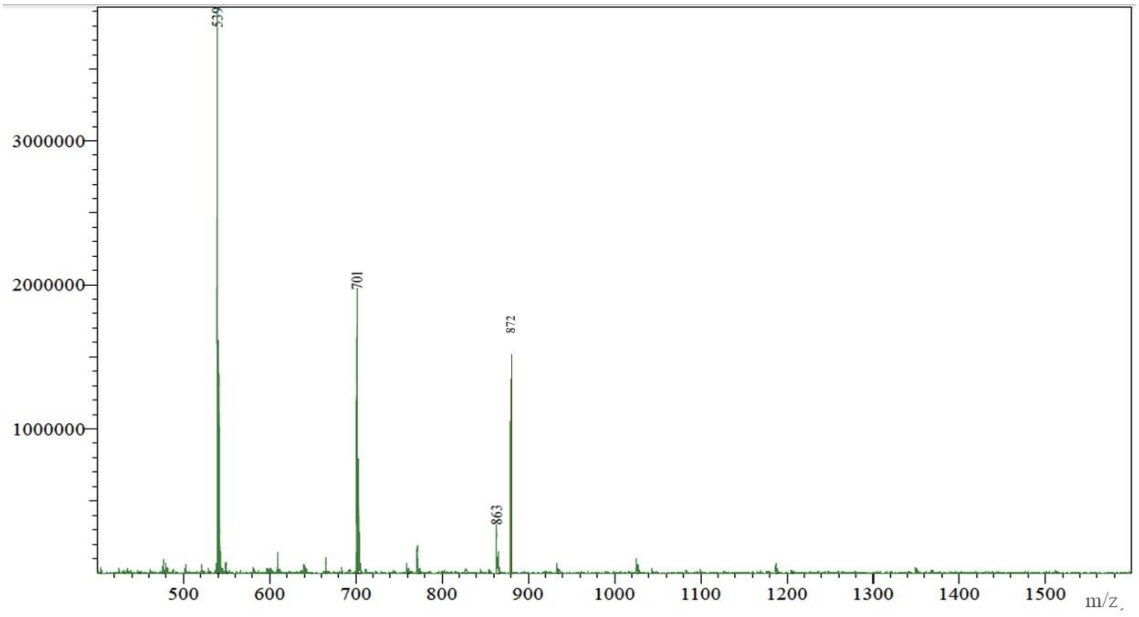

4?----Line#:4 R.Time:----(Scan#:----)
MassPeaks:837
Spectrum Mode:Averaged 1.12-1.19(68-72) Base Peak:407(22731)
BG Mode:Calc Segment 1 - Event 2

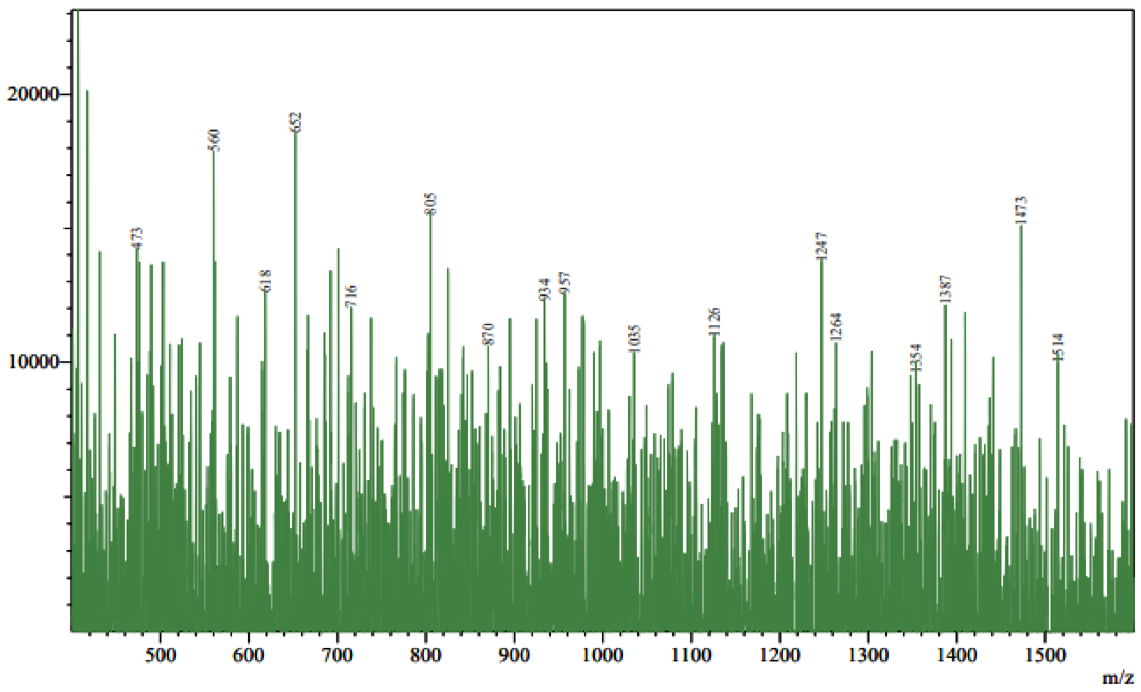

5?----Line#:5 R.Time:----(Scan#:----)
MassPeaks:569
Spectrum Mode:Averaged 7.72-7.79(462-466) Base Peak:581(113759)
BG Mode:Calc Segment 1 - Event 2

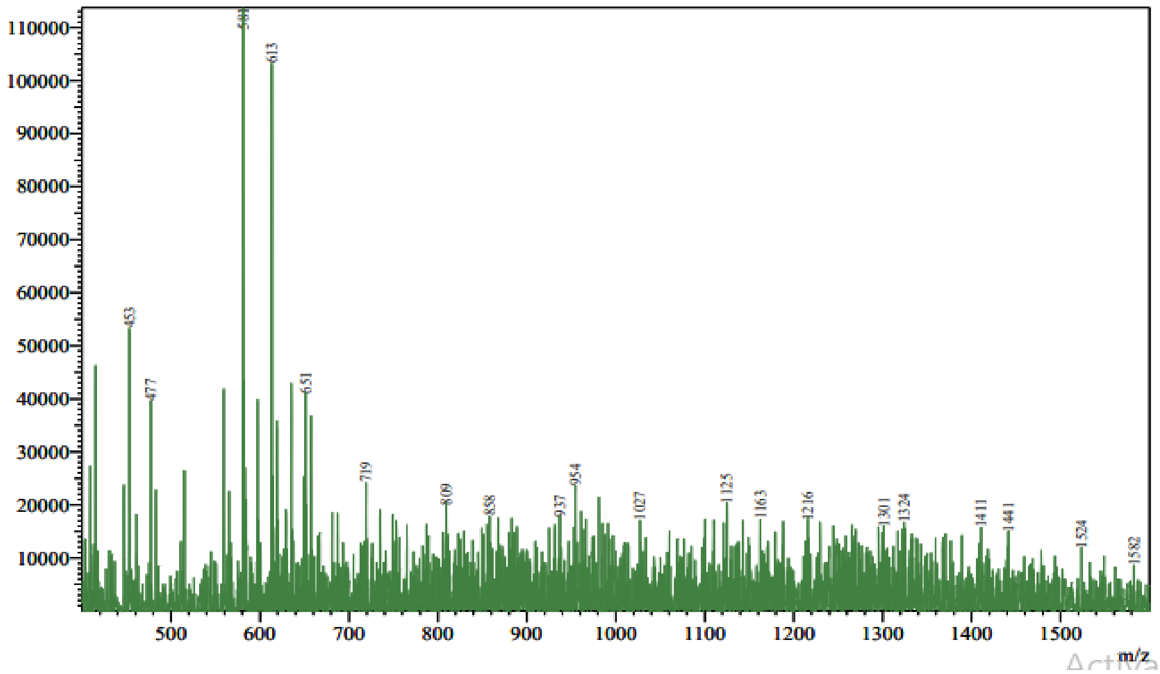

6?----Line#:6   R.Time:----(Scan#:----)
MassPeaks:210
Spectrum Mode:Averaged 8.49-8.56(508-512) Base Peak:593(157319)
BG Mode:Calc Segment 1 - Event 2

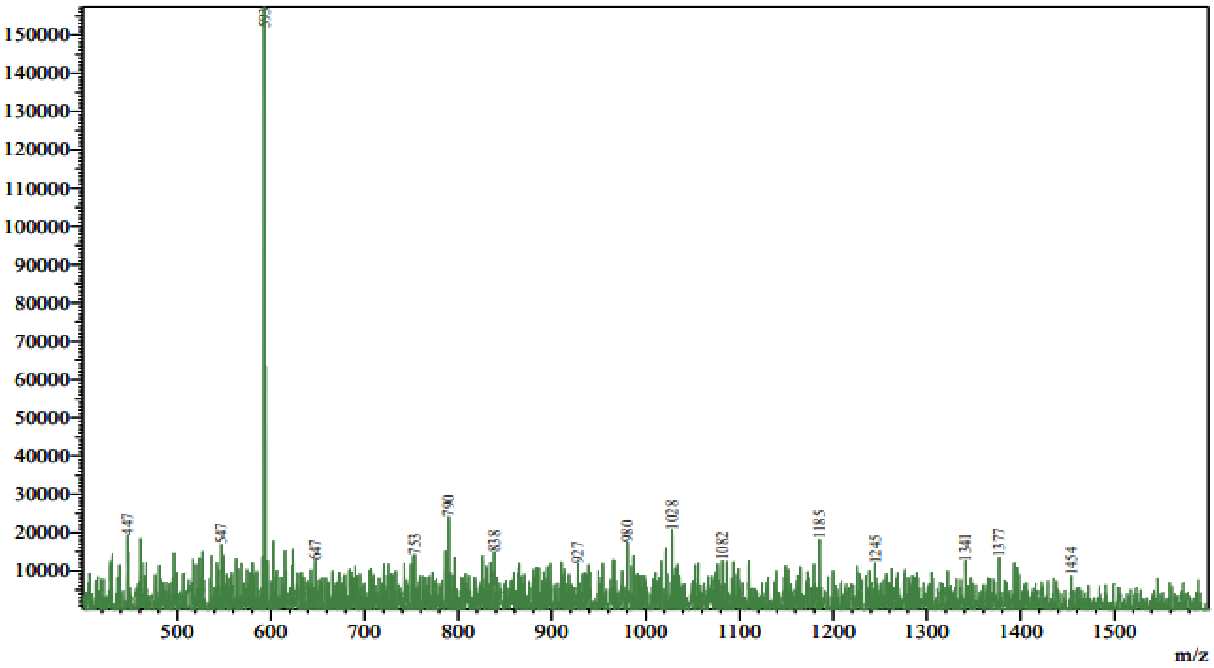

Peak Table

PDA Ch1 254nm

Peak#	Ret. Time	Area	Height	Lambda max
1	0.62	973951	304135	652/482/570/681/753
2	0.70	378448	96711	651/256/276/239/338
3	0.80	445266	88685	275/651/788/776/466
4	0.89	919988	78783	284/662/487/407/775
5	1.59	4611	290	267/662/642
6	2.13	345061	20890	263/705
7	2.79	12551	906	268
8	3.72	2759	183	676
9	5.71	301796	12281	267/487/656/466/646
10	6.18	23264	3101	268/245/487/656/466
11	6.40	72849	5382	277/244/486/466/444
12	6.61	21793	3633	231/266/346/486/466
13	6.80	60263	4067	233/327/486/466/655
14	6.98	55375	6205	233/348/486/466/655
15	7.24	58009	6618	228/327/486/466/655
16	7.44	20118	2959	236/327/486/466/477
17	7.62	78157	11002	227/486/466/477/542
18	7.73	30080	5387	233/348/486/466/477
19	7.94	66333	8217	237/327/485/654/631
20	8.42	4822	690	243/267/327/485/653
21	8.50	5091	780	244/270/327/485/653
22	8.72	8818	1496	267/327/662/484/569
23	8.90	1519	444	239/327/652/484/569
24	8.99	6281	1229	238/327/662/568/496
25	9.09	3101	652	239/327/652/662/568
26	9.42	2991	380	244/327/484/568/662
27	9.80	45550	6351	239/269/327/652/484
28	10.26	2997	611	244/347/272/652/568
29	10.56	54568	5537	237/348/568/477/496
30	11.02	9707	1737	244/273/344/652/484
31	11.11	7156	1233	244/273/346/652/484
32	11.24	5248	760	243/274/348/652/568
33	11.39	3582	452	243/273/652/484/567
34	11.80	17168	1399	242/652/484/567/496
35	12.02	11145	1627	241/275/652/483/568
36	12.17	50534	3494	240/274/652/483/567
37	14.87	1082351	19103	233/275/652/483/567
38	17.25	9821831	51204	229/275/652/420/483
39	23.14	76320	1652	249/410/427/652/483
40	25.23	48392	2319	251/410/652/483/496
41	26.81	589692	11705	248/410/427/331/652
42	27.22	305026	12893	248/410/427/331/652
43	28.67	2341067	27441	245/409/426/652/503
Total		18375630	814624	

PDA Ch2 200-400nm

Peak#	Ret. Time	Area	Height	Lambda max
1	0.09	1068	223	672/705
2	0.62	5802556	1588305	652/482/570/681/753
3	0.77	2649095	482586	259/652/483/409/566
4	0.88	7480101	369751	284/657/488/425/467
5	2.12	106319	8388	263/642
6	2.77	12328	956	265/639
7	3.13	7445	244	677
8	3.52	5089	471	686
9	3.75	28057	1738	
10	4.35	13475	877	
11	4.80	35250	1443	487/656
12	5.42	25624	1722	226/487/656/467/587
13	5.71	180791	13393	267/487/656/466/646
14	6.35	16641	2738	236/287/486/466/656
15	6.41	11850	2876	277/244/486/466/444
16	6.61	5697	1368	231/266/345/328/486
17	6.80	41909	3750	233/327/422/486/466
18	6.98	72665	12850	233/348/486/466/655
19	7.24	95154	21944	228/327/486/466/655
20	7.45	4674	1166	235/327/486/466/655
21	7.62	572468	75693	227/486/466/477/542
22	7.93	257102	25768	237/327/485/654/631
23	8.41	127610	8010	243/267/327/485/571
24	8.72	66319	6851	267/327/662/484/569
25	8.99	96588	10167	238/327/652/662/496
26	9.10	69219	7581	239/327/652/662/476
27	9.38	66602	4741	244/331/484/568/662
28	9.60	28140	4502	244/652/484/568/496
29	9.70	33360	6740	241/354/484/568/662
30	9.80	130142	12758	239/269/327/652/484
31	10.26	47912	3857	244/347/272/652/568
32	10.56	239639	20009	237/348/568/476/496
33	11.02	41919	4501	244/273/345/652/484
34	11.11	25340	4194	244/273/346/652/484

Peak#	Ret. Time	Area	Height	Lambda max
35	11.25	36527	4137	243/273/347/652/568
36	11.39	36280	4108	243/273/652/354/484
37	11.81	122405	5877	242/652/484/567/496
38	12.18	243802	11934	240/274/652/483/375
39	17.23	146313062	795647	229/275/652/420/483
40	23.14	108572	2378	249/410/652/483/496
41	25.23	45764	2298	251/410/652/483/496
42	26.81	607387	12521	248/410/427/331/652
43	27.21	322193	13659	248/410/427/652/496
44	28.67	2618776	35762	245/409/426/652/503
Total		168852915	3600481	

Method

<<Header>>
Generated : 6/22/2021 3:24:57 PM
GeneratedBy : System Administrator
Modified : 10/26/2022 11:24:01 AM
ModifiedBy : System Administrator

<<System Controller>>
Model : LC-2040 Controller
Event1 : Off
Event2 : Off
Event3 : Off
Event4 : Off
Degassing Unit : On
Sample Load Timing : Off

<<Data Acquisition>>
LC Stop Time : 35.00 min
PDA Detector Name : PDA
PDA Sampling Frequency : 4.16667 Hz
PDA Start Time : 0.00 min
PDA End Time : 35.00 min
PDA Time Constant : 0.480 sec

<<Pump>>
Mode : Low pressure gradient
Pump A : LC-2040 Pump
Flow : 0.2000 mL/min
B Conc. : 10.0 %
C Conc. : 0.0 %
D Conc. : 0.0 %
B Curve : 0
C Curve : 0
D Curve : 0
PressMax : 66.0 MPa
PressMin : 0.0 MPa
LPGE Mode : Auto
Compressibility Setting : Off
Gradient Start Adjustment : None

<<Autosampler>>
Autosampler Model : LC-2040 Autosampler
Enable Autosampler : Use
Rinsing Volume : 500 uL
Rinsing Speed : 35 uL/sec
Sampling Speed : 5.0 uL/sec
Purge Time : 2.0 min
Rinse Mode : Before and after aspiration
Rinse Dip Time : 0 sec
On Time Injection : Off
Cooler Temperature : 15 C
Rack Plate L Temperature Control : Off
Rack Plate R and Ctrl Rack Temperature Control : Off
Air Gap Volume : Off

<<Sample Pretreatment>>
Mode : Standard

<<Oven>>
Oven Model : LC-2040 Oven
Enable Oven : Not Used

<<LC Time Program>>

Time	Module	Command	Value	Comment
0.01	Pumps	B.Conc	10	
2.00	Pumps	B.Conc	10	
5.00	Pumps	B.Conc	30	
15.00	Pumps	B.Conc	70	
22.00	Pumps	B.Conc	80	
25.00	Pumps	B.Conc	90	
26.00	Pumps	B.Conc	90	
29.00	Pumps	B.Conc	10	
30.00	Pumps	B.Conc	10	
35.00	Controller	Stop		

<<Mobile Phase Name>>
Mobile Phase A : Water
Mobile Phase B : Acetonitrile

<<Auto Purge>>

<<PDA>>
PDA Model : LC-2030/2040 PDA Detector
Lamp : D2
Start Wavelength : 200 nm
End Wavelength : 800 nm

Use Cell Temp.	: Use
Cell Temp.	: 40 C
Slit Width	: 8 nm
Polarity	: +
Spectrum Resolution	: 512
Maximum Data Size	: 128 MB
Reference Correction	: Not Used
Channel 1 Wavelength	: 254 nm
Channel 1 Bandwidth	: 4 nm
Channel 1 Output Range	: 1.0 AU/V
Channel 1 Polarity	: +
Channel 2 Wavelength	: 254 nm
Channel 2 Bandwidth	: 4 nm
Channel 2 Output Range	: 1.0 AU/V
Channel 2 Polarity	: +

<<MS Parameter>>

CID Gas	:Not Used
Conversion Dynode	:2.50 kV
Initial Valve Position	:-
--Segment 1 Event 1--	
Acquisition Mode	:Q3 Scan
Polarity	:Positive
Start Time	:0.000 min
End Time	:35.000 min
Compound Name	:
Start m/z	:400.00
End m/z	:1600.00
Scan Speed	:1250 u/sec
Event Time	:1.000 sec
Q3 Resolution	:Unit
Interface Volt.	:4.00 kV
DUIS Corona Needle Volt.	:4.50 kV
DL Bias	:Use the Data in the Tuning File
Qarray Bias	:Use the Data in the Tuning File
Q3 Prerod Bias	:Use the Data in the Tuning File
--Segment 1 Event 2--	
Acquisition Mode	:Q3 Scan
Polarity	:Negative
Start Time	:0.000 min
End Time	:35.000 min

Compound Name	:
Start m/z	:400.00
End m/z	:1600.00
Scan Speed	:1250 u/sec
Event Time	:1.000 sec
Q3 Resolution	:Unit
Interface Volt.	:4.00 kV
DUIS Corona Needle Volt.	:4.50 kV
DL Bias	:Use the Data in the Tuning File
Qarray Bias	:Use the Data in the Tuning File
Q3 Prerod Bias	:Use the Data in the Tuning File
Automatic Exclude Period	:10.00 sec
Exclude Isotope Range(Begin)	:0.00 u
Exclude Isotope Range(End)	:2.00 u
Minimum Iteration Times	:3
Tolerance	:0.50 u

<<MS Program>>

<<Analog Table>>

<<Interface>>

Interface	:ESI
Interface heater	:On
Interface Temperature	:300 C
Desolvation Temperature	:526 C
DL Temperature	:250 C
Nebulizing Gas Flow	:3.00 L/min
Heating Gas	:On
Heating Gas Flow	:10.00 L/min
Heat Block	:400 C
Drying Gas	:On
Drying Gas Flow	:10.00 L/min

<<Peak Integration>>

<PDA>

Channel	: Extracted Chromatogram
Algorithm	: Chromatopac
Width	: 5 sec
Slope	: 1000 uV/min
Drift	: 0 uV/min
T.DBL	: 1000 min
Min.Area/Height	: 1000 counts
Calculated by	: Area
Auto	: Off
Register Spectrum to Table	: Off
Max Slices	: 0
Peak Top Detection	: Normal

RT Compensation Mode : Fine
Tailing Off : Off
Min.Area/Height is made effective in Manual Integration : Off
Noise Calculation Settings : Noise Data : Current Data
Calculation Method : ASTM
Range : Whole Range
Interval : 0.5 min
Include the Peak Detected Range : Off
Detection Limit Coefficient : 3.3
Quantitative Limit Coefficient : 10.0
Drift Calculation Settings : 0.00 - 15.00 min

Channel : Ch1 254nm,4nm
Algorithm : Chromatopac
Width : 5 sec
Slope : 1000 uV/min
Drift : 0 uV/min
T.DBL : 1000 min
Min.Area/Height : 1000 counts
Calculated by : Area
Auto : Off
Register Spectrum to Table : Off
Max Slices : 0
Peak Top Detection : Normal
RT Compensation Mode : Fine
Tailing Off : Off
Min.Area/Height is made effective in Manual Integration : Off
Noise Calculation Settings : Noise Data : Current Data
Calculation Method : ASTM
Range : Whole Range
Interval : 0.5 min
Include the Peak Detected Range : Off
Detection Limit Coefficient : 3.3
Quantitative Limit Coefficient : 10.0
Drift Calculation Settings : 0.00 - 15.00 min

Channel : Ch2 200-400nm,4nm
Algorithm : Chromatopac
Width : 5 sec
Slope : 1000 uV/min
Drift : 0 uV/min
T.DBL : 1000 min
Min.Area/Height : 1000 counts
Calculated by : Area
Auto : Off
Register Spectrum to Table : Off
Max Slices : 0

Peak Top Detection : Normal
RT Compensation Mode : Fine
Tailing Off : Off
Min.Area/Height is made effective in Manual Integration : Off
Noise Calculation Settings : Noise Data : Current Data
Calculation Method : ASTM
Range : Whole Range
Interval : 0.5 min
Include the Peak Detected Range : Off
Detection Limit Coefficient : 3.3
Quantitative Limit Coefficient : 10.0
Drift Calculation Settings : 0.00 - 15.00 min

<<Integration Time Program(Method)>>
<PDA>
Channel : Extracted Chromatogram
Time Program : None

Channel : Ch1 254nm,4nm
Time Program : None

Channel : Ch2 200-400nm,4nm
Time Program : None

<<Integration Time Program(Data)>>
<PDA>
Channel : Extracted Chromatogram
Time Program : None

Channel : Ch1 254nm,4nm
Time Program : None

Channel : Ch2 200-400nm,4nm
Time Program : None

<<Identification>>
<PDA>
Window/Band : Window
Window : 5.00 %
Identification Method : Absolute

(S6): Identification of abamectin produced by the isolate St.53 using LC-MS with standard

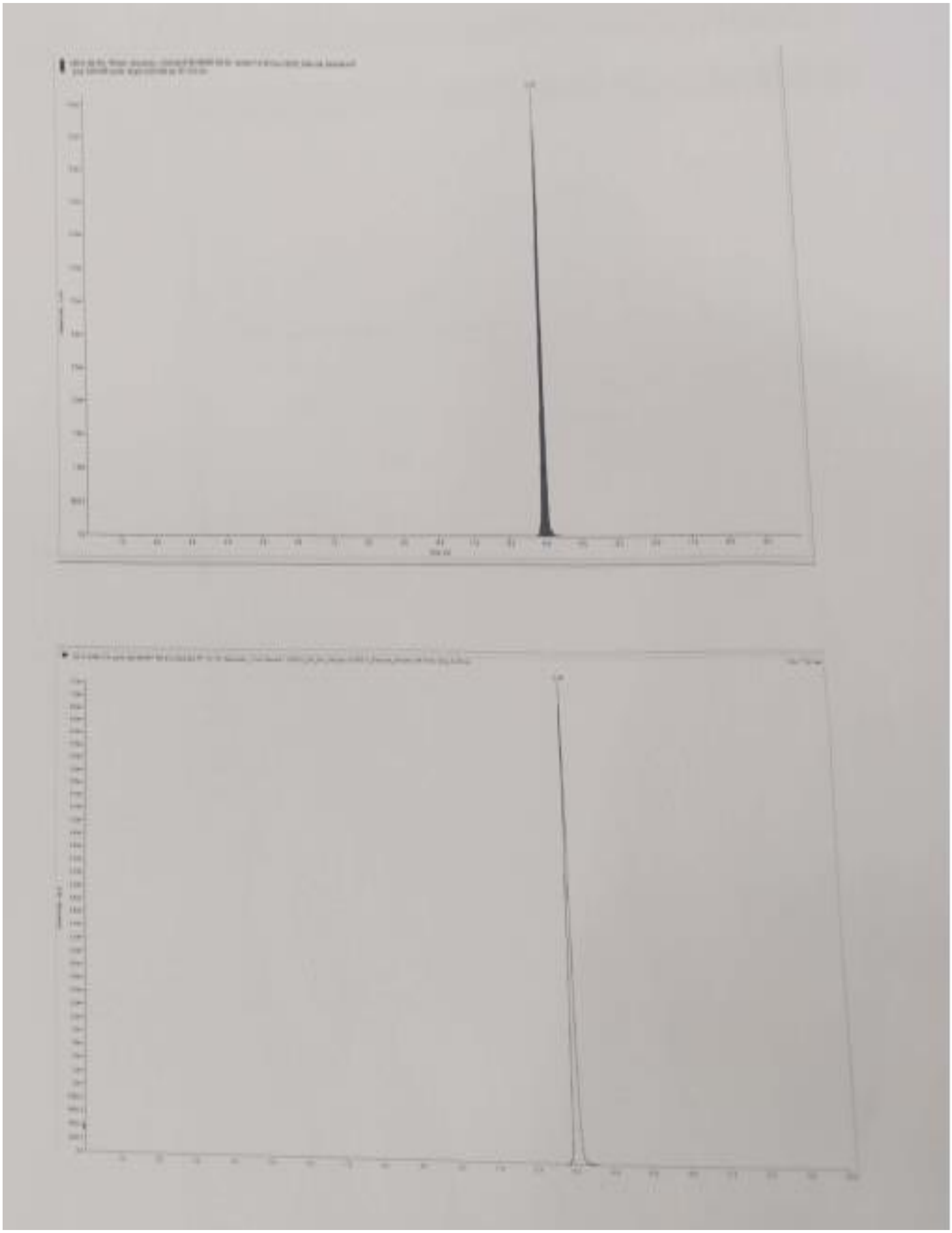

Abamectin

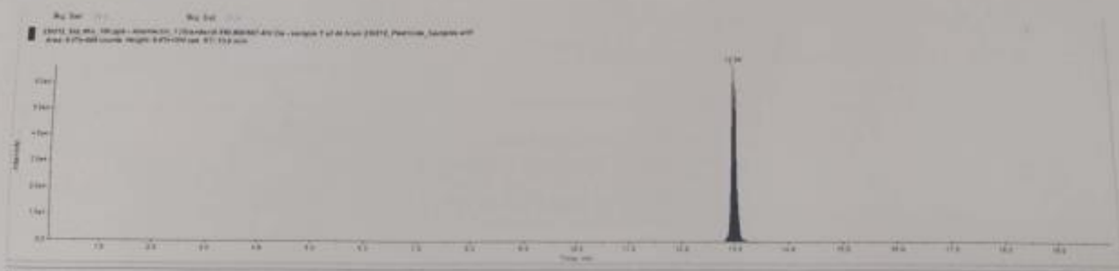

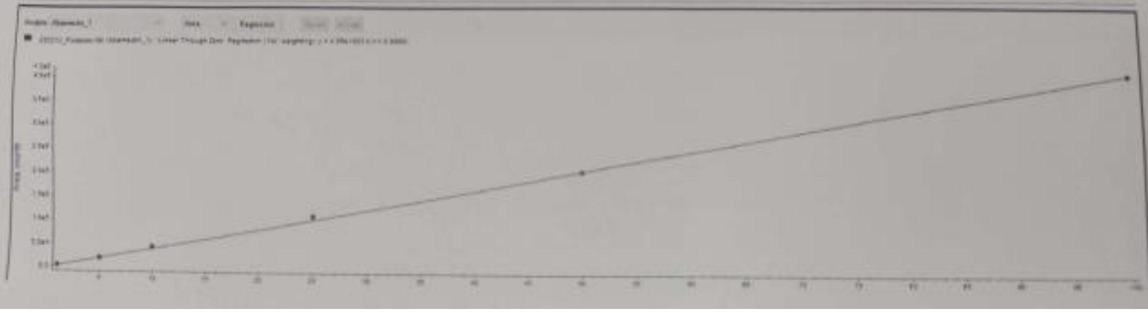

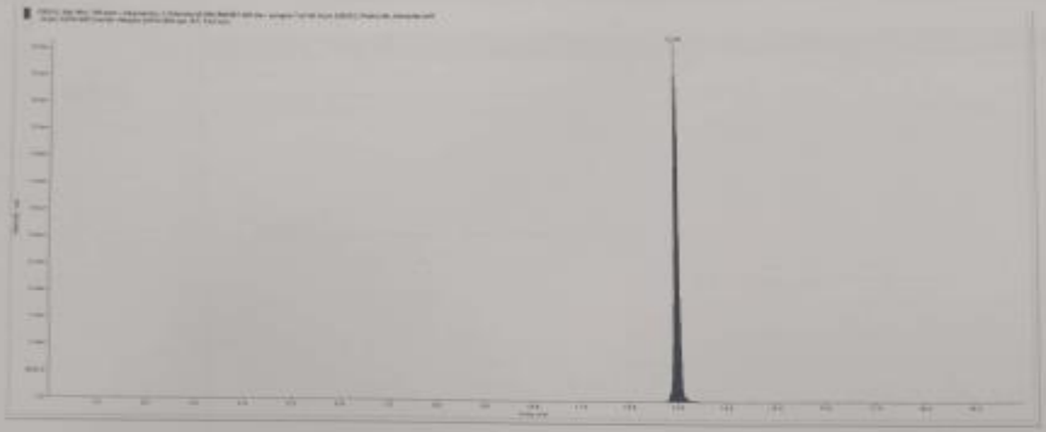

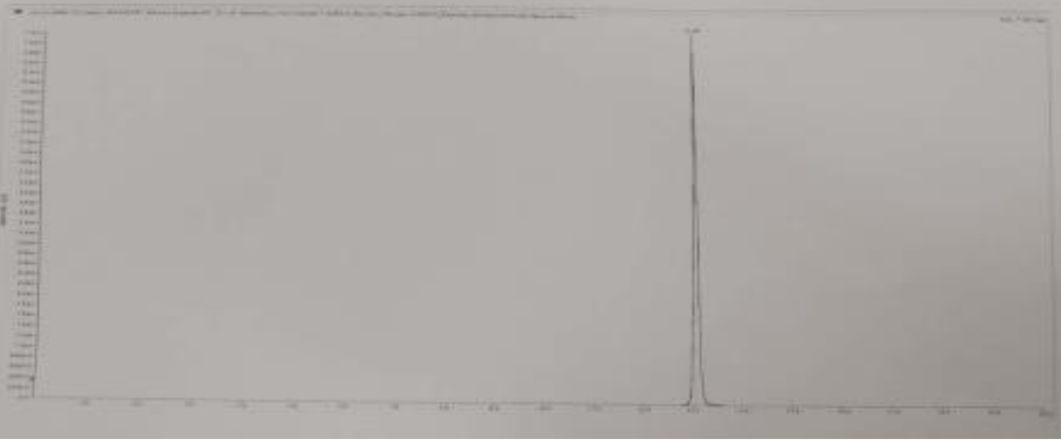

	Abamectin Mas Parametars							
CXQ1		Q3	RT	ID	DP	EP	CE	CXP
880.5		567.4	15.5	Abamectin_1	81	10	19	8
880.5		305.3	15.5	Abamectin_2	81	10	37	16

LC Parametars		
Mobile phase	A(Aques)	10% Ammoniom Formate/Water
	B(Organic)	0% Ammoniom Formate/Methan

Mobile phase Gradiant			
Flow	0.4Ml/Min		
	Time	A	B
	0	100%	
	15		100%
	18		100%
	18.05	100%	
	20	100%	

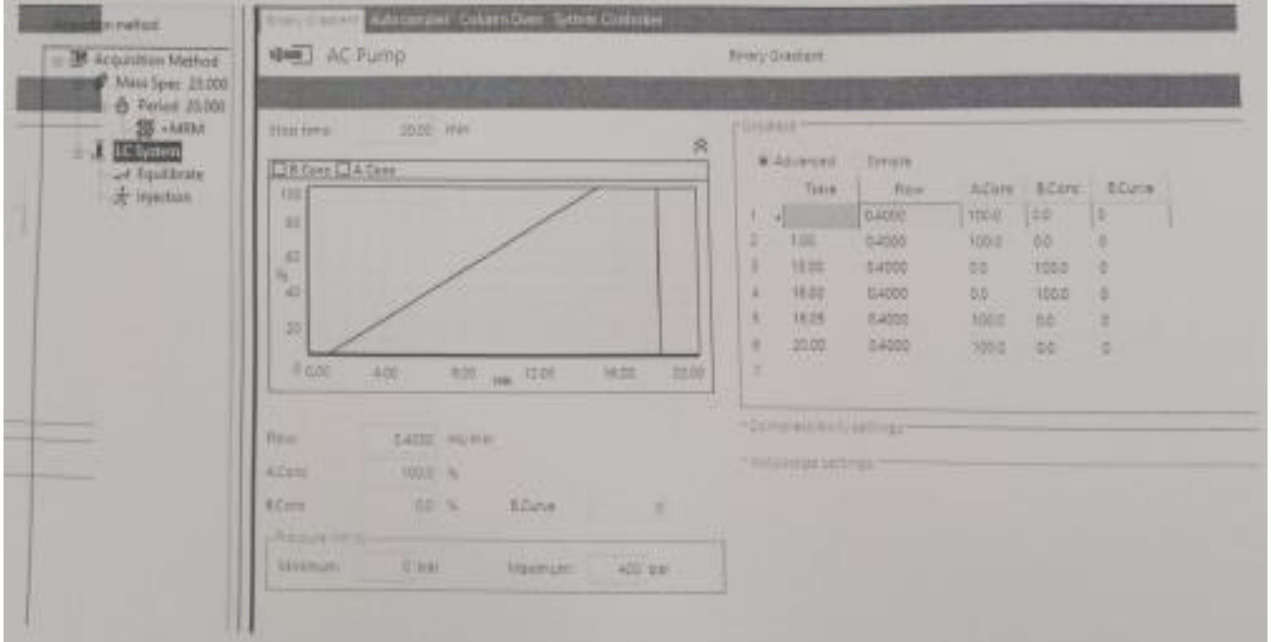

Supplement: Supplementary file 3 — Supplementary Material 3 [file 12866_2024_3466_MOESM3_ESM.pdf]
